# Proprotein convertase subtilisn/kexin type 9 inhibitors and small interfering RNA therapy for cardiovascular risk reduction: A systematic review and meta-analysis

**DOI:** 10.1371/journal.pone.0295359

**Published:** 2023-12-06

**Authors:** Tasnim F. Imran, Ali A. Khan, Phinnara Has, Alexis Jacobson, Stephanie Bogin, Mahnoor Khalid, Asim Khan, Samuel Kim, Sebhat Erqou, Gaurav Choudhary, Karen Aspry, Wen-Chih Wu

**Affiliations:** 1 Providence VA Medical Center, Providence, Rhode Island, United States of America; 2 Lifespan Cardiovascular Institute, Rhode Island and Miriam Hospitals, Providence, Rhode Island, United States of America; 3 Warren Alpert Medical School of Brown University, Providence, Rhode Island, United States of America; 4 Northwestern University, Evanston, Illinois, United States of America; 5 Weil Cornell College of Medicine, New York, New York, United States of America; Catholic University of Brasilia, BRAZIL

## Abstract

**Background:**

Atherosclerotic cardiovascular disease (ASCVD) is the leading cause of mortality worldwide. Atherosclerosis occurs due to accumulation of low-density lipoprotein cholesterol (LDL-c) in the arterial system. Thus, lipid lowering therapy is essential for both primary and secondary prevention. Proprotein convertase subtilisn/kexin type 9 (PCSK9) inhibitors (Evolocumab, Alirocumab) and small interfering RNA (siRNA) therapy (Inclisiran) have been demonstrated to lower LDL-c and ASCVD events in conjunction with maximally tolerated statin therapy. However, the degree of LDL-c reduction and the impact on reducing major adverse cardiac events, including their impact on mortality, remains unclear.

**Objective:**

The purpose of this study is to examine the effects of PCSK9 inhibitors and small interfering RNA (siRNA) therapy on LDL-c reduction and major adverse cardiac events (MACE) and mortality by conducting a meta-analysis of randomized controlled trials.

**Methods:**

Using Pubmed, Embase, Cochrane Library and clinicaltrials.gov until April 2023, we extracted randomized controlled trials (RCTs) of PCSK9 inhibitors (Evolocumab, Alirocumab) and siRNA therapy (Inclisiran) for lipid lowering and risk of MACE. Using random-effects models, we pooled the relative risks and 95% CIs and weighted least-squares mean difference in LDL-c levels. We estimated odds ratios with 95% CIs among MACE subtypes and all-cause mortality. Fixed-effect model was used, and heterogeneity was assessed using the I^2^ statistic.

**Results:**

In all, 54 studies with 87,669 participants (142,262 person-years) met criteria for inclusion. LDL-c percent change was reported in 47 studies (n = 62,634) evaluating two PCSK9 inhibitors and siRNA therapy. Of those, 21 studies (n = 41,361) included treatment with Evolocumab (140mg), 22 (n = 11,751) included Alirocumab (75mg), and 4 studies (n = 9,522) included Inclisiran (284mg and 300mg). Compared with placebo, after a median of 24 weeks (IQR 12–52), Evolocumab reduced LDL-c by -61.09% (95% CI: -64.81, -57.38, p<0.01) and Alirocumab reduced LDL-c by -46.35% (95% CI: -51.75, -41.13, p<0.01). Inclisiran 284mg reduced LDL-c by -54.83% (95% CI: -59.04, -50.62, p = 0.05) and Inclisiran 300mg reduced LDL-c by -43.11% (95% CI: -52.42, -33.80, p = 0.01). After a median of 8 months (IQR 6–15), Evolocumab reduced the risk of myocardial infarction (MI), OR 0.72 (95% CI: 0.64, 0.81, p<0.01), coronary revascularization, 0.77 (95% CI: 0.70, 0.84, p<0.01), stroke, 0.79 (95% CI: 0.66, 0.94, p = 0.01) and overall MACE 0.85 (95% CI: 0.80, 0.89, p<0.01). Alirocumab reduced MI, 0.57 (0.38, 0.86, p = 0.01), cardiovascular mortality 0.35 (95% CI: 0.16, 0.77, p = 0.01), all-cause mortality 0.60 (95% CI: 0.43, 0.84, p<0.01), and overall MACE 0.35 (0.16, 0.77, p = 0.01).

**Conclusion:**

PCSK9 inhibitors (Evolocumab, Alirocumab) and siRNA therapy (Inclisiran) significantly reduced LDL-c by >40% in high-risk individuals. Additionally, both Alirocumab and Evolocumab reduced the risk of MACE, and Alirocumab reduced cardiovascular and all-cause mortality.

## Introduction

Atherosclerotic cardiovascular disease (ASCVD) is the leading cause of death worldwide, contributing to one in three deaths globally [1, 2]. Atherosclerosis occurs due to accumulation of low-density lipoprotein cholesterol (LDL-c) in the arterial system. Acute rupture of atherosclerotic plaque can lead to myocardial infarction or stroke [3]. LDL-c is one of the major modifiable risk factors for development and progression of ASCVD. Thus, lipid lowering is essential to treatment of this condition. However, despite widespread use of statin therapies, 80% of patients do not reach the guideline recommended LDL-c targets [4]. The American Heart Association / American College of Cardiology (AHA/ACC) guidelines and the European Society of Cardiology / European Atherosclerosis Society (ESC/EAS) guidelines recommend statins as first-line therapies with ezetimibe as a second-line option for those intolerant to statins or unable to achieve target LDL-c levels [5, 6]. If further reduction in LDL-c is needed, proprotein convertase subtilisn/kexin type 9 (PCSK9) inhibitors (Evolocumab, Alirocumab) or small interfering RNA (siRNA) therapy (Inclisiran) can be added in a step-wise approach [7]. Furthermore, the 2023 European of Society Acute Coronary Syndrome guidelines state that in cases where a patient is already on the highest tolerable dose of a statin and had LDL-c levels that suggest that targets are unlikely to be met with statin monotherapy, combination therapy may be initiated at the outset with statin and ezetimibe during a hospitalization for acute coronary syndrome. The 2018 American Heart Association/ American College of Cardiology guideline and the 2017 National Lipid Association update suggest two treatment thresholds for use of PCSK9 inhibitors for high-risk ASCVD: LDL-c ≥ 70 mg/dL or non-high-density lipoprotein cholesterol (non-HDL-C) ≥ 100 mg/dL after maximally tolerated LDL-c lowering therapies [5, 8].

PCSK9 inhibitors, such as Evolocumab and Alirocumab, bind to circulating PCSK9 and prevent it from interacting with LDLR (LDL receptor). This allows more LDLR to be available on the surface of liver cells, leading to more efficient removal of LDL cholesterol. Inclisiran is a small interfering RNA (siRNA) molecule that binds to and degrades the mRNA that encodes for PCSK9, preventing the production of the PCSK9 protein [7]. This leads to an increase in the number of LDL receptors on the surface of liver cells, which in turn leads to an increase in the clearance of LDL cholesterol from the bloodstream. While PCSK9 inhibitors and siRNA therapy have been shown to lower LDL cholesterol levels, studies vary on the degree of LDL reduction. In addition, there is some uncertainty about the effects of these therapies on adverse outcomes, particularly mortality. Thus, the purpose of our study is to examine the effect of PCSK9 inhibitors and siRNA therapies on major adverse cardiac events and degree of LDL reduction in patients with cardiovascular disease and those at high risk of cardiovascular disease. We performed a systematic review and meta-analysis that quantitatively evaluates the impact of these therapies on clinical outcomes and LDL-c levels.

## Methods

**Fig 1:**
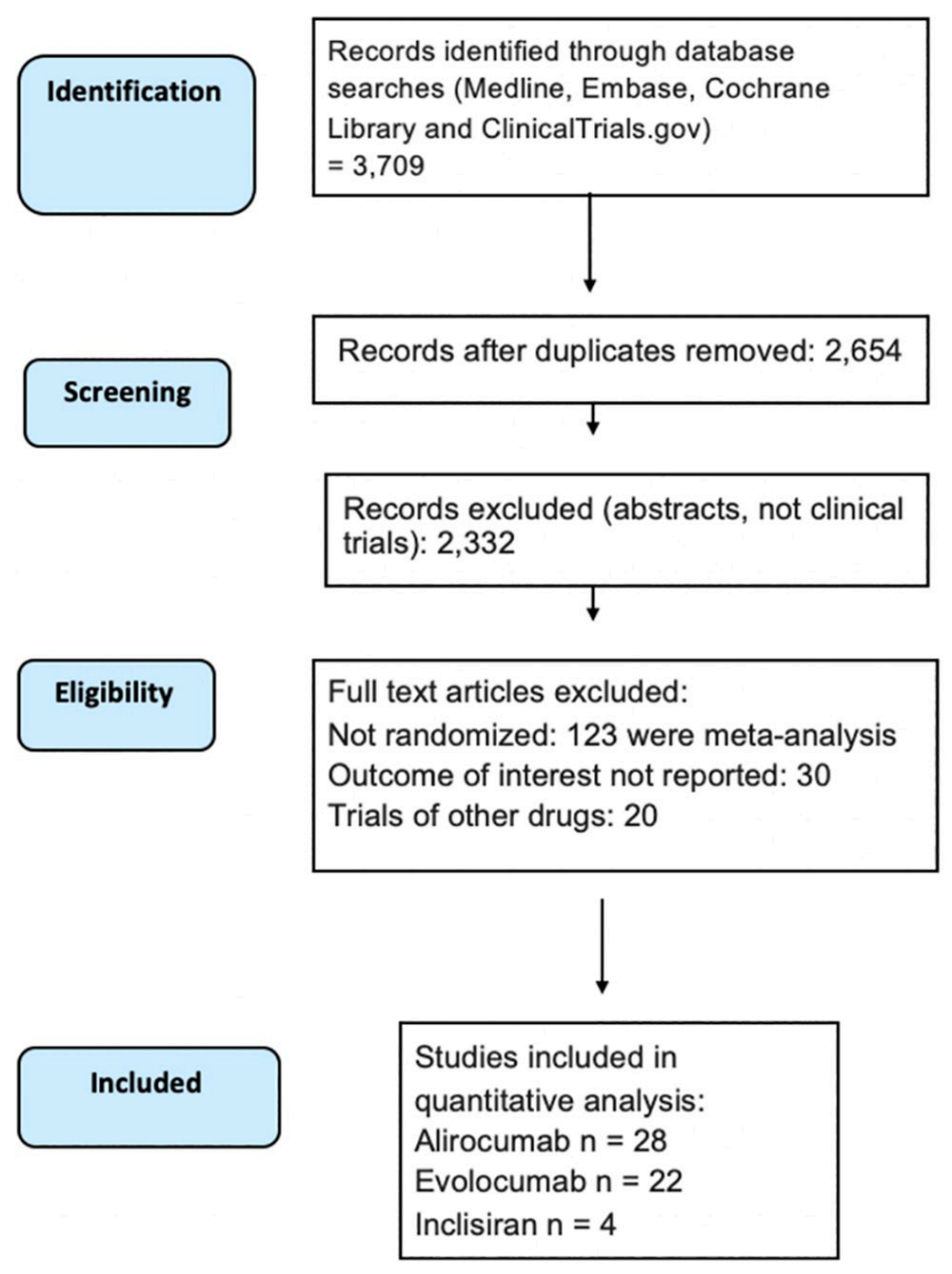
Process of study selection according to PRISMA guidelines. The detailed definition of each MACE endpoint is included in the Supplemental Section (data in S1 Text in S2 File).

Studies were eligible for inclusion if PCSK9 inhibitors, Evolocumab or Alirocumab, or siRNA therapy, Inclisiran^®^, were administered in a randomized controlled trial with measurement of LDL-cholesterol levels and/or assessment of clinical outcomes including cardiovascular mortality, all-cause mortality. Studies were excluded if they did not have a sufficient number of events to allow for extraction of effect measures for each clinical outcome. In cases where several studies from the same cohort were available, data from the study with the latest follow-up were selected. We only included randomized controlled trials with five months of follow-up or longer. We excluded literature reviews, cross-sectional studies, preclinical studies, animal studies, and effect estimates from conference abstracts when a full published study was not available. We conducted the meta-analysis according to the Preferred Reporting Items for Systematic Reviews and Meta-Analyses (PRISMA) checklist. Two independent reviewers screened and extracted data from full-text articles and Cohen’s kappa was calculated to determine interrater reliability. The process of study selection is detailed in Fig 1 (PRISMA diagram). We used a combination of subject headings and search terms related to PCSK9 inhibitors, siRNA therapy, CVD mortality, mortality, myocardial infarction and stroke.

We performed a literature search for all relevant publications using comprehensive databases: PubMed, Ovid MEDLINE, Ovid Embase, Web of Science, the Wiley Cochrane Library, the Cochrane Database, and Google Scholar databases up to April 2023. We used the following terms for MEDLINE: (Alirocumab [MeSH Terms] OR Evolocumab [All Fields] OR Inclisiran [All Fields] OR PCSK9 inhibitors OR small interfering RNA therapy [All Fields] OR Proprotein convertase subtilisin/kexin type 9 inhibitor [All Fields] AND cardiovascular outcomes [MeSH terms] OR myocardial infarction [All Fields] OR stroke [All Fields] OR coronary heart disease [All Fields] OR coronary revascularization[All Fields] OR mortality [All Fields] OR all-cause mortality [All Fields] OR cardiovascular mortality [All Fields] OR CVD mortality [All Fields] OR cardiac mortality [All Fields] OR heart failure [All Fields]) (S1 Table in S2 File). These search terms were also used for EMBASE, Web of Science, and Google Scholar. To identify any additional studies not captured by these searches, we also hand searched reference lists of primary and review articles. In addition, we searched proceedings of relevant societies (American Heart Association, American College of Cardiology, European Society of Cardiology, National Lipid Association).

We obtained the full-text articles of the relevant studies and conducted a detailed review to ensure that they met the inclusion criteria for the meta-analysis. Using a standardized data extraction form to collect relevant data from each of the included studies, we extracted information on study design, sample size, patient characteristics, intervention details, and outcome measures. The following variables were extracted from each study using a standardized data extraction template: title, authors, year of publication, name of study cohort, geographic location, age, percentage of men/women, body mass index, follow-up time, sample size, number of participants, follow-up time, mean and standard deviation of LDL-c levels, covariates adjusted for in the multivariable analysis, and effect measures with their 95% CIs of mortality, CVD mortality, myocardial infarction, coronary revascularization, stroke, heart failure risk. The variable extraction was performed independently by two reviewers and cross-checked.

### Statistical analysis

Descriptive statistics for pooled data such as year of publication, sample size, percentage of women and follow-up period are described. The studies were evaluated with regards to similarity of baseline patient characteristics, methods, and duration of follow-up. Random-effects models were used to synthesize the weighted pooled least-squares mean difference in LDL cholesterol levels with 95% confidence intervals. Some studies reported standard errors while others reported 95% confidence intervals; in these cases, the lower and upper limit of the confidence intervals were calculated using the reported standard errors. The number of positive and negative MACE were defined among treatment groups (Evolocumab, Alirocumab, and Inclisiran) compared to placebo. Among Evolocumab and Alirocumab studies, death (all-cause) was reported, and MACE was stratified by type: stroke, myocardial infarction, coronary revascularization, heart failure, and cardiovascular mortality.

Using the meta program in STATA, we generated forest plots and determined the group-specific and overall I^2^ statistic. The fixed-effects or random-effects models were chosen for each outcome based on the between-study heterogeneity [9]. We used the Cochran Q test and the I^2^ statistic to evaluate for heterogeneity between studies. The I^2^ was calculated as follows using Stata: I^2^ = Q -(k-1)/Q x 100%, where k is the number of studies, and k-1 is the degrees of freedom. We predefined high heterogeneity as an I^2^ statistic greater than 50%. Fixed-effect models were used for I^2^ <50% and random-effects models were used for I^2^>50%. We estimated odds ratios with 95% confidence intervals (CI) among MACE subtypes and mortality. We performed meta-regression adjusting for age and percent women using the stata meta regress function.

### Quality assessment

We used the Cochrane Collaboration’s tool to assess risk of bias and the quality of randomized controlled trials included in the meta-analyses (performed independently by two reviewers) [10]. This tool assesses six domains of potential bias: selection bias, performance bias, detection bias, attrition bias, and reporting bias. To evaluate the certainty of evidence, we used the Grading of Recommendations, Assessment, Development and Evaluation (GRADE) framework developed by the GRADE working group [11]. This included the following aspects: overall risk of bias, degree of imprecision, consistency of results, potential for publication bias, and extent of indirectness. To assess for publication bias, we inspected funnel plots and performed the Egger’s regression test using the metafunnel and metabias commands. All analyses were performed using Stata 17.0 (StataCorp, College Station, Texas); all tests were two sided, and a p value <0.05 was considered statistically significant.

## Results

In total, 54 studies with 87,669 participants (142,262.29 person-years) met criteria for analysis (Fig 1). Of these, 47 studies (n = 62,634) reported LDL-c reduction, CV morality was reported in 18 studies (n = 48,121), of which 11 included Evolocumab, 5 included Alirocumab, and 2 included Inclisiran. HF was reported in 9 studies (n = 39,300), in which 3 included Evolocumab and 6 included Alirocumab. Myocardial infarction was reported in 20 studies (n = 46,126), in which 9 included Evolocumab, 9 included Alirocumab, and 2 included Inclisiran. Coronary revascularization was reported in 14 studies (n = 38,577), in which 7 included Evolocumab and 7 included Alirocumab. Stroke was reported in 14 studies (n = 44,946), in which 6 included Evolocumab, 6 included Alirocumab, and 2 included Inclisiran. All-cause mortality was examined in 24 studies (n = 50,830), in which 5 included Evolocumab and 19 studies included Alirocumab. Cohen’s kappa for interrater reliability was 0.94. In all, 40.5% of participants were women. The follow-up median was 24 weeks (interquartile range: 12 to 52 weeks; S2 Table in S2 File). Table 1 shows the baseline summary population characteristics across the studies by drug type. Mean age was 60–63 years, 33–41% of participants were women, and 86–91% identified as White.

**Table 1:** Summary of demographic characteristics of study participants across drug types.

	Evolocumab	Alirocumab	Inclisiran
	N = 21 studies	N = 22 studies	N = 4 studies
Age (mean ± SD)	59.88 ± 11.62	60.77 ± 10.11	62.7 ± 10.07
Female (%)	41%	39.4%	32.8%
Race			
White	85.6%	87%	90.7%
Non-White	14.4%	13%	8.3%
Body mass index (kg/m2)	28.5	29.7	-
Comorbidities (%)			
Hypertension	60.3%	62.8%	85.2%
Smoker	25.5%	16.8%	15.7%
Diabetes mellitus	20.2%	28.6%	47.5%
History of Heart Failure	19%	14.4%	-
Prior Stroke	9.5%	5.8%	-
Prior MI	25%	17.8%	-
Coronary Artery Disease	22.6%	49.1%	-

– indicates that data was not available

### LDL-c reduction

**Fig 2:**
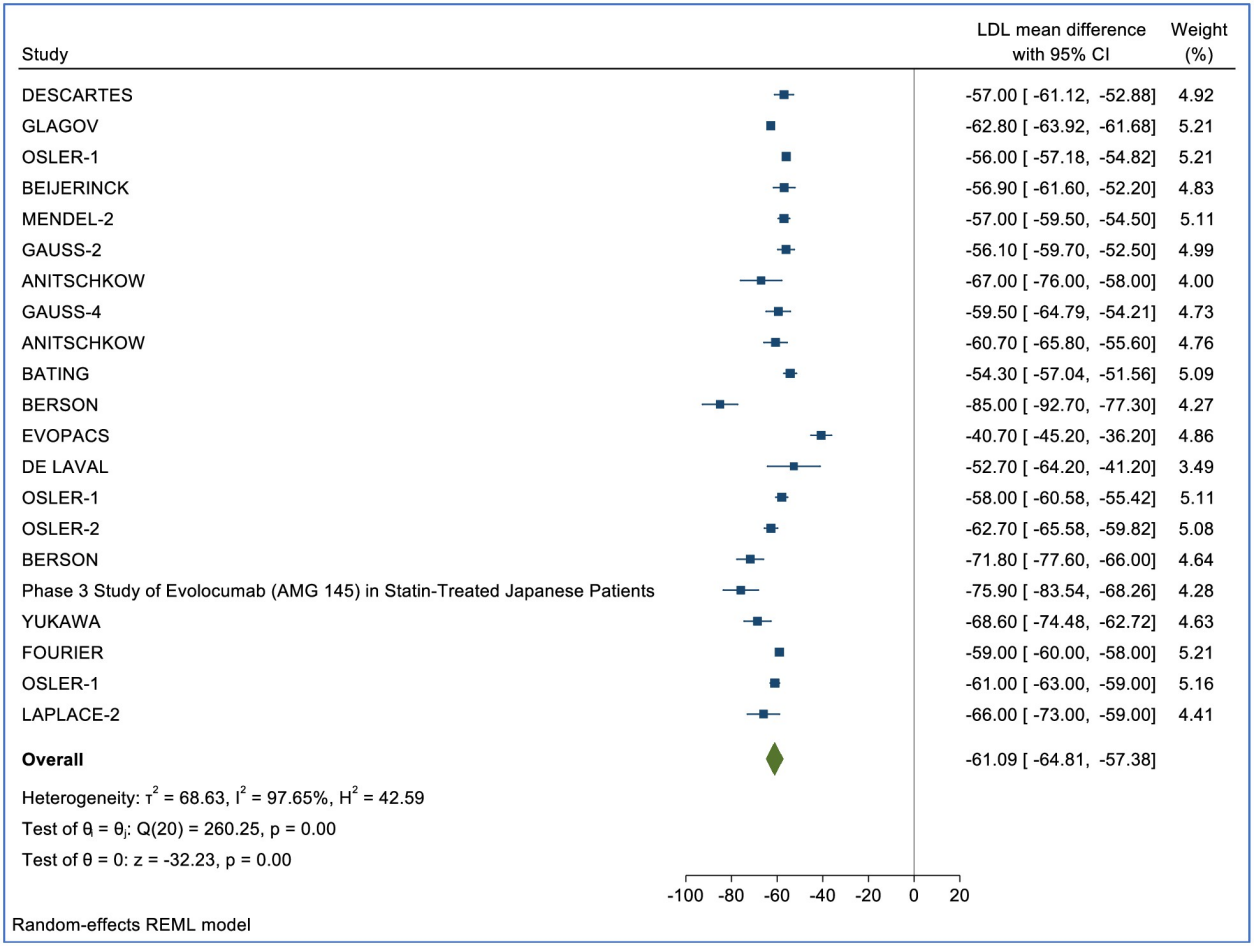
Forest plot for the percentage change in LDL-c mean difference for Evolocumab.

**Fig 3:**
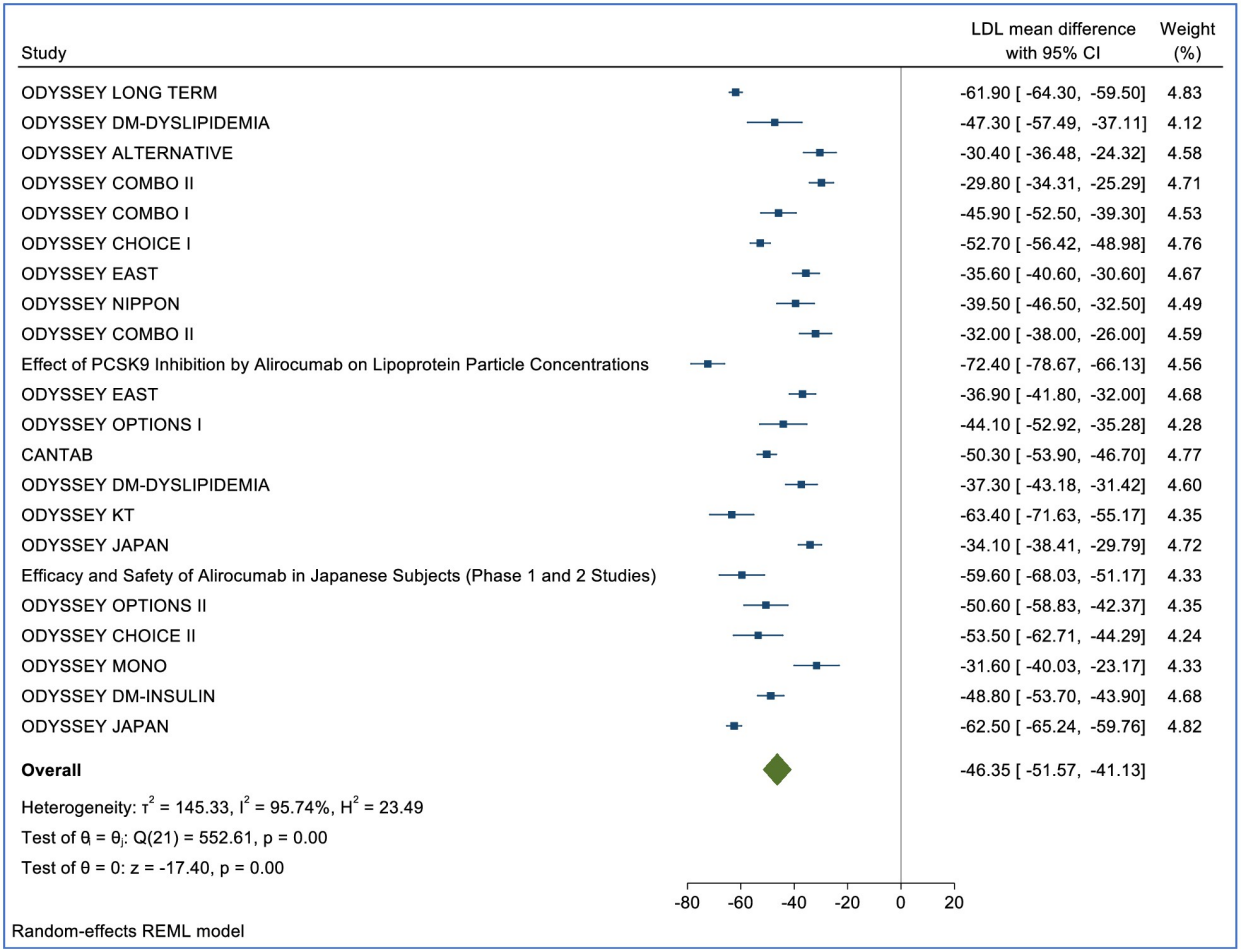
Forest plot for the percentage change in LDL-c mean difference for Alirocumab.

**Fig 4:**
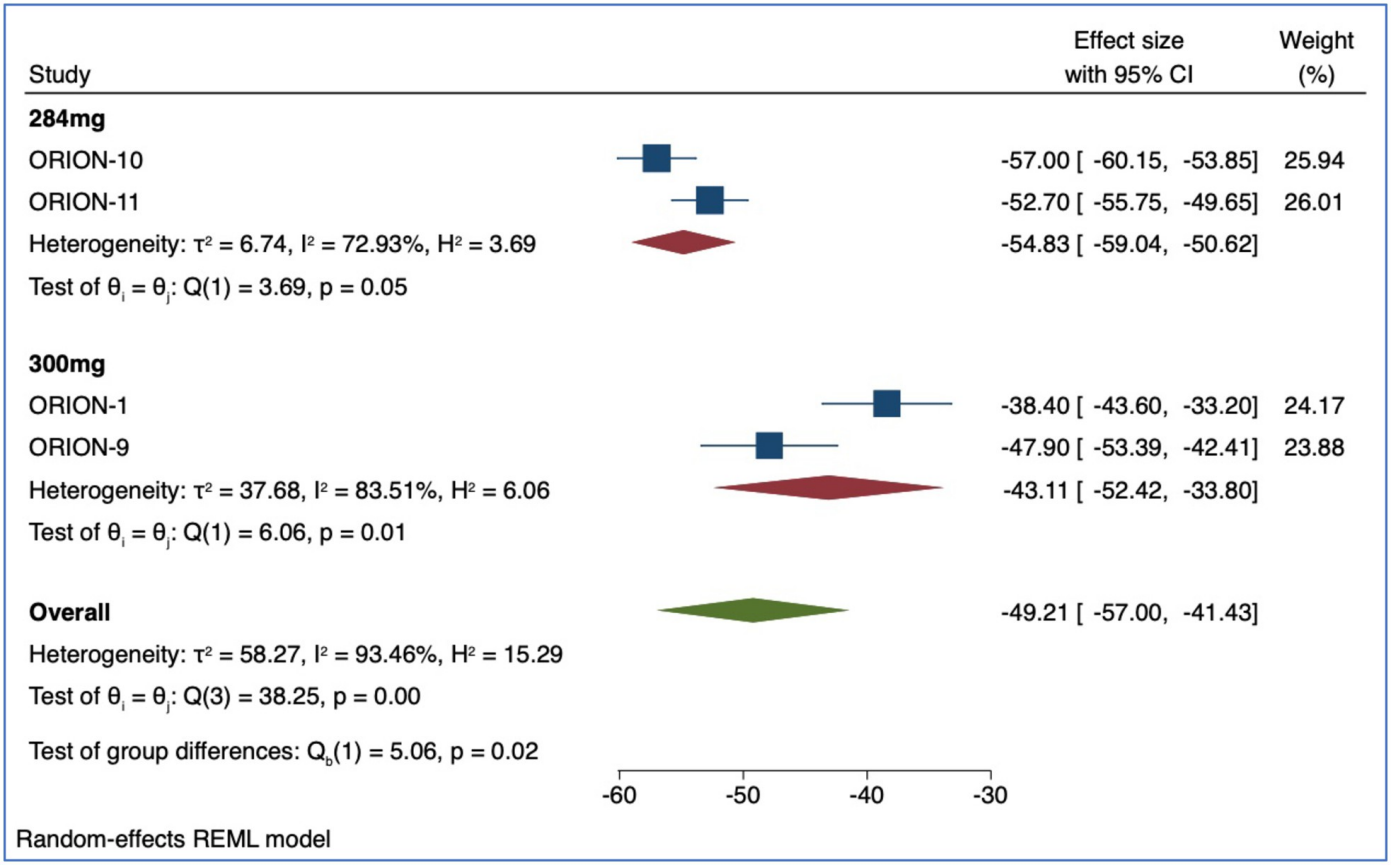
Forest plot for the percentage change in LDL-c mean difference for Inclisiran (284mg, 300mg).

LDL-c percent change was reported in 47 (RCTs) (n = 62,634) for Alirocumab, Evolocumab and Inclisiran. Of those, 21 studies (n = 41,361) included treatment with Evolocumab (140mg), 22 (n = 11,751) included Alirocumab (75mg), and 4 studies (n = 9,522) included Inclisiran (284mg and 300mg). Compared with placebo, after a median of 24 weeks (IQR 12–52), Evolocumab reduced LDL-c by -61.09% (95% CI: -64.81, -57.38, p<0.01) (Fig 2). Compared with placebo, Alirocumab reduced LDL-c by -46.35% (95% CI: -51.75, -41.13, p<0.01) (Fig 3). Compared with placebo, Inclisiran 284mg reduced LDL-C by -54.83% (95% CI: -59.04, -50.62, p = 0.05) and Inclisiran 300mg reduced LDL-c by -43.11% (95% CI: -52.42, -33.80, p = 0.01) (Fig 4). Overall, all three therapies significantly reduced LDL cholesterol compared to placebo (Table 2).

**Table 2:** Percent change of LDL-c with PCSK9 inhibitors and siRNA therapy versus control.

Drug	N	Mean Difference	95% CI	P-value
Evolocumab	41361	-61.09	-64.81 to -57.38	<0.001
Alirocumab	11751	-46.35	-51.57 to -41.13	<0.001
Inclisiran	9522	-49.21	-57 to -41.43	<0.001

### Clinical outcomes

**Fig 5:**
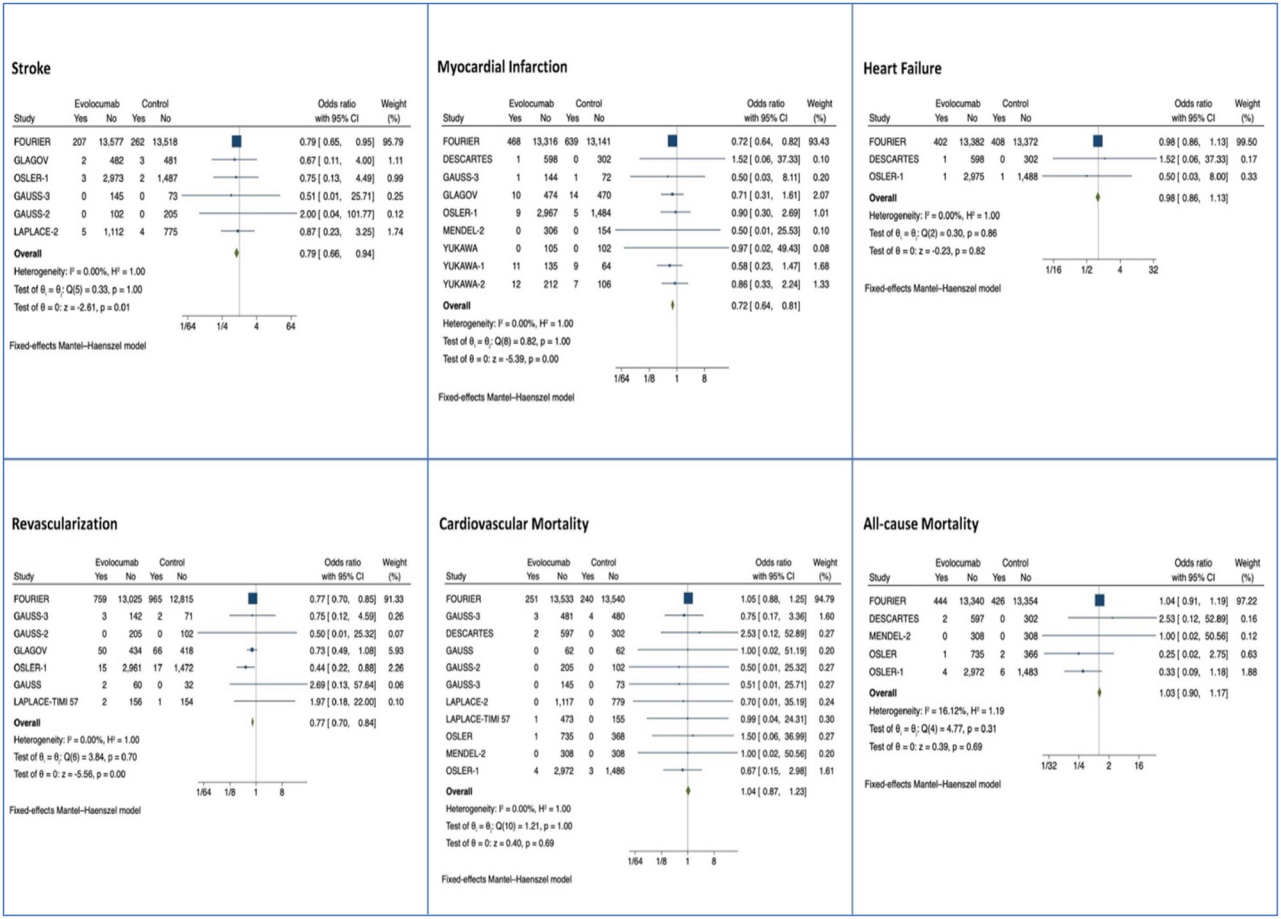
Forest plots of Evolocumab MACE by subtype (stroke, MI, heart failure, coronary revascularization, cardiovascular mortality, and all-cause mortality).

**Fig 6:**
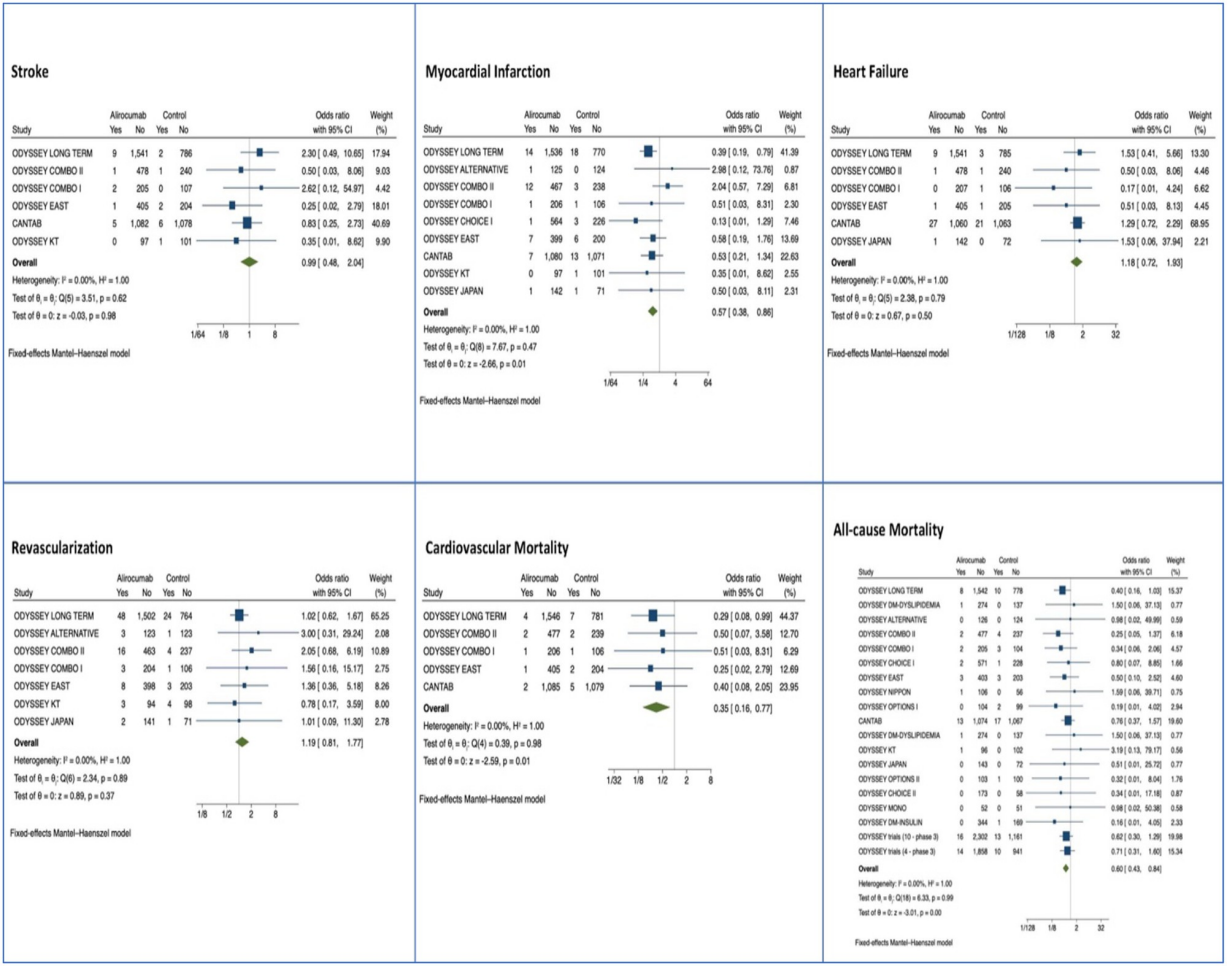
Forest plots of Alirocumab MACE by subtype (stroke, MI, heart failure, coronary revascularization, cardiovascular mortality, and all-cause mortality).

**Fig 7:**
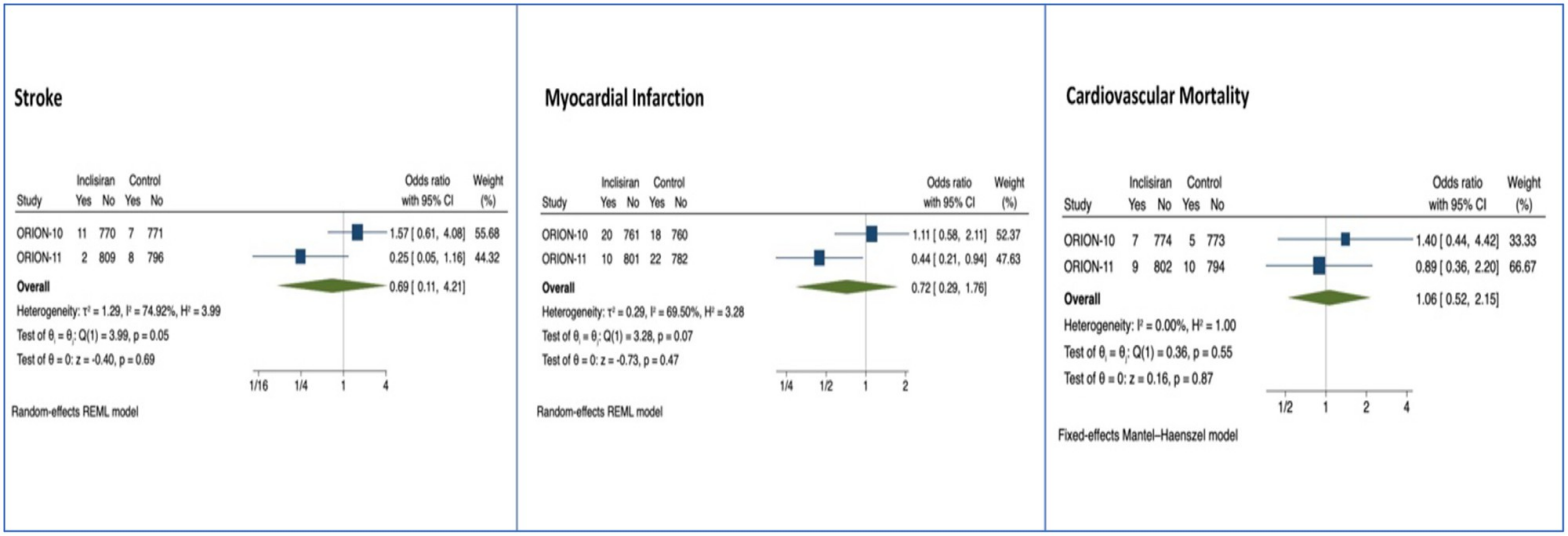
Forest plots of Inclisiran MACE by subtype (stroke, MI, and cardiovascular mortality).

Cardiovascular (CV) mortality, heart failure (HF), myocardial infarction (MI), revascularization, and stroke were major adverse cardiovascular events assessed for Evolocumab and Alirocumab studies. Only CV mortality, MI, and stroke in the short term were assessed for Inclisiran (284mg) studies. After a median of 8 months (IQR 6–15), Evolocumab reduced the risk of myocardial infarction (MI), OR 0.72 (95% CI: 0.64, 0.81, p<0.01), coronary revascularization, OR 0.77 (95% CI: 0.70, 0.84, p<0.01), stroke, OR 0.79 (95% CI: 0.66, 0.94, p = 0.01) and overall major adverse cardiac events, OR 0.85 (95% CI: 0.80, 0.89, p<0.01) (n = 42,637) (Fig 5). Alirocumab reduced myocardial infarction, OR 0.57 (0.38, 0.86, p = 0.01), cardiovascular mortality OR 0.35 (95% CI: 0.16, 0.77, p = 0.01), all-cause mortality OR 0.60 (95% CI: 0.43, 0.84, p<0.01), and overall major adverse cardiac events, OR 0.35 (0.16, 0.77, p = 0.01) (Fig 6) (n = 15,760). Among the Inclisiran treatment group, there were no significant differences in the overall risk of major adverse cardiac events compared with the control group (odds ratio [OR] -0.21, 95% CI -1.11, to 0.69, p > 0.05), however there is a smaller clinical trial sample size and number of major adverse cardiac events for Inclisiran, as well as lack of long-term data (Fig 7).

Using meta regression adjusting for age and percent women, we found no significant difference in heterogeneity (S6 Table in S2 File).

### Quality assessment

For quality assessment, we used the Cochrane tool for the assessment of bias. The assessment was performed independently by two reviewers. Most included information was from randomized clinical trials at found to be at low risk of bias. To evaluate the certainty of evidence, we used the Grading of Recommendations, Assessment, Development and Evaluation (GRADE) framework developed by the GRADE working group, which revealed high certainty for Alirocumab vs placebo and Evolocumab vs placebo [11]. There were no direct comparisons of Evolocumab and Alirocumab with the outcome of mortality, thus the quality of evidence for this comparison was low. Inspection of the funnel plot for Evolocumab and Alirocumab trials did not suggest publication bias (Egger’s test p value 0.71) (S1 Fig in S2 File). However, the funnel plots and Egger’s test (p = 0.046) for Inclisiran suggests evidence of publication bias (S2 Fig in S2 File).

## Discussion

This meta-analysis, including 54 studies totaling 87,669 participants, found that PCSK9 inhibitors and siRNA therapy led to a significant reduction in LDL-c. Evolocumab, Alirocumab, and Inclisiran were found to have a >40% reduction in LDL-c compared to placebo (Table 1). Furthermore, Evolocumab and Alirocumab demonstrated a significant reduction in risk of MACE, and Alirocumab reduced cardiovascular and all-cause mortality. Inclisiran did not appear to have significant MACE risk-reduction, however further clinical trials for Inclisiran are lacking, therefore follow-up data for MACE is insufficient at this time. These results support the efficacy of PCSK9 inhibitors and siRNA therapy in reducing LDL-c, and PCKS9 inhibitors in decreasing risk of adverse cardiovascular events.

The results of our study align with overall previous findings in reduction of LDL-cholesterol levels and MACE, with additional new insights. Previous meta-analysis reported that Alirocumab reduced the risk of overall MACE (RR 0.85, 95% CI 0.78–0.93), stroke (RR 0.76, 95% CI 0.60–0.96), and coronary revascularization (RR 0.88, 95% CI 0.80–0.96) [12]. Most prior studies did not find a significant difference in all-cause mortality with PCSK9 inhibitors [13–15]. While our study found an overall reduction in MACE with Alirocumab, we also observed reductions in the risk of myocardial infarction, cardiovascular mortality, and all-cause mortality. This is likely due to the inclusion of new randomized trials that have been published since the previous studies were conducted, and longer follow-up periods.

A prior meta-analysis demonstrated a reduction of -54.95 (95% CI -59.09, -50.81) in LDL-cholesterol levels for Evolocumab and -48.99 (-54.28, -43.71) for Alirocumab. Our findings for Evolocumab and Alirocumab also align with these results, with reductions of -61.09% (-64.81, -57.38) and -46.35% (-51.75, -41.13), respectively [16]. Additionally, a large randomized trial with a mean follow-up of 9 months also found an overall LDL reduction of 54.6% with PCSK9 inhibitors, similar to our findings [17]. Similar to our results, a previous meta-analysis found that Evolocumab reduced the risk of MACE (RR 0.80, 95% CI 0.75–0.85), myocardial infarction (RR 0.73, 95% CI 0.65–0.82), stroke (RR 0.79, 95% CI 0.66–0.95), and coronary revascularization (RR 0.78, 95% CI 0.72–0.86). Another study revealed that Alirocumab reduced all-cause mortality (RR 0.83, 95% CI 0.72–0.95), whereas Evolocumab (RR 1.04, 95% CI 0.91, 1.18) and Inclisiran did not show significant associations (RR 1.00, 95% CI 0.58, 1.72) [18]. This was similar to our study in that Alirocumab was found to reduce cardiovascular mortality OR 0.35 (95% CI: 0.16, 0.77) and all-cause mortality OR 0.60 (95% CI: 0.43, 0.84).

When compared to statins, studies have shown that the benefits of statin therapy are limited in patients who exhibit resistance or intolerance to high-dose statins. However, the addition of PCSK9 inhibitors to statin therapy can result in a remarkable 50–60% greater reduction in LDL-c levels compared to statin therapy alone. The FOURIER trial demonstrated that patients receiving maximally tolerated statin therapy and randomized to receive Evolocumab achieved a substantial 59% reduction in LDL-c levels after 48 weeks, compared to the placebo group. Consistent findings regarding LDL-c reductions have been reported in other clinical trials [19].

Furthermore, statins have been found to reduce the risk of cardiovascular events in both women (relative risk [RR], 0.81 [95% CI, 0.74–0.89]) and men (RR, 0.82 [95% CI, 0.78–0.85]). However, statins did not demonstrate a significant reduction in all-cause mortality in women compared to men (RR, 0.92 [95% CI, 0.76–1.13] vs. RR, 0.79 [95% CI, 0.72–0.87]), nor in stroke incidence (RR, 0.92 [95% CI, 0.76–1.10] vs. RR, 0.81 [95% CI, 0.72–0.92]) [20]. When used as an adjunct to high-dose statins, PCSK9 inhibitors have been shown to reduce overall cardiovascular events [18, 21].

The mechanisms by which PCSK9 inhibitors reduce adverse cardiovascular events and mortality are multifactorial, involving the reduction of LDL cholesterol levels, anti-inflammatory effects, and improvement of endothelial function [18]. When PCSK9 binds to LDL receptors on the surface of hepatocytes, it promotes their degradation. This leads to a decrease in the uptake of LDL-c by the liver and an increase in the levels of circulating LDL-c, which is associated with an increased risk of major cardiovascular events [22]. However, PCSK9 inhibitors reduce the levels of PCSK9 in the bloodstream, resulting in a decrease in this risk. They also decrease levels of other lipids, such as apolipoprotein B, lipoprotein (a), and non-HDL-C. Additionally, PCSK9 inhibitors have been shown to have anti-inflammatory effects and to improve endothelial function, which can also contribute to their cardiovascular benefits. PCSK9 inhibitors have been shown to reduce inflammation markers such as C-reactive protein and interleukin-6. Furthermore, they can improve endothelial function by enhancing the production of nitric oxide, a molecule that dilates blood vessels and improves blood flow. Even though both PCSK9 monoclonal antibody (Alirocumab or Evolocumab) and Inclisiran reduce LDL-c concentrations by increasing LDL receptors and decreasing active PCSK9 protein levels, their mechanisms of action differ. Monoclonal antibodies function outside the cells by binding and blocking circulating PCSK9 protein, but they do not prevent PCSK9 production inside the cells. In contrast, Inclisiran works inside the cells by inhibiting the translation of PCSK9 messenger RNA, leading to a decrease in both intracellular and plasma PCSK9 levels. Treatment with Inclisiran thus has a potential advantage of longer duration of its lipid-lowering effect compared to PCSK9 monoclonal antibodies. It is administered through subcutaneous injections once every six months, while PCSK9 monoclonal antibodies need to be injected once every 2–4 weeks.

Our study has limitations. First, the quality of the included studies can affect the validity of the findings for some outcomes. Alirocumab demonstrated greater effectiveness in reduction of cardiovascular and all-cause mortality compared to placebo, which was not noted with Evolocumab. However, these outcomes are subject to imprecision and heterogeneity because direct head-to-head studies comparing Alirocumab and Evolocumab with these outcomes are lacking. Second, meta-analysis relies on the availability of data from multiple studies, which was not always comprehensive. For example, differences in patient populations or treatment protocols can make it difficult to combine data from different studies, thus increasing the heterogeneity among the studies. Another limitation is that of publication bias. The chance of this was low in the current meta-analysis as most included studies were sufficiently powered and well-executed randomized clinical trials. The funnel plot and Egger’s test confirmed low risk of bias overall. However, not all studies were sufficiently powered for every clinical outcome. For instance, some trials were lacking information on particular adverse outcomes with insufficient numbers of events. Some studies had small sample sizes or short follow-up periods. As for Inclisiran, there have not been many studies conducted on its effectiveness in reducing the risk of major adverse cardiac events.

Despite the aforementioned limitations, our study has several strengths. First, our meta-analysis included 54 studies (87,669 participants and 142,262.29 person-years) which allows for adequate power to detect modest associations. Second, studies included in this meta-analysis were conducted at various geographic locations, thus, the overall effect may be generalizable. In addition, all studies used a standardized blood tests to measure LDL-c as well as standard definitions of clinical outcomes, minimizing measurement bias of the outcomes. Additionally, we used a robust strategy following the PRISMA guidelines, explicit inclusion criteria, and grading of evidence and publication bias.

Additional robust clinical trials are needed to fully evaluate the impact of Alirocumab on cardiovascular and all-cause mortality and of Inclisiran on major adverse cardiovascular outcomes in the long-term.

## Supporting information

S1 Checklist PRISMA checklist. (DOCX)

S1 File Raw dataset. (DOCX)

S2 File Supplemental tables. (DOCX)
