# Supplementary material for: Proprotein convertase subtilisn/kexin type 9 inhibitors and small interfering RNA therapy for cardiovascular risk reduction: A systematic review and meta-analysis
Source: PLoS One. 2023 Dec 6;18(12):e0295359. doi: 10.1371/journal.pone.0295359 (PMC10699593; doi:10.1371/journal.pone.0295359)
Supplement: S1 File — (DOCX) [file pone.0295359.s002.docx]

**LDL Mean Difference for Evolocumab**

| **Study** | **LDL Mean Difference** | **Lower 95% CI Limit** | **Upper 95% CI Limit** |
| --- | --- | --- | --- |
| DESCARTES | -57.00 | -61.12 | -52.88 |
| GLAGOV | -62.80 | -63.92 | -61.68 |
| OSLER-1 | -56.00 | -57.18 | -54.82 |
| BEUJERINCK | -56.90 | -61.60 | -52.20 |
| MENDEL-2 | -57.00 | -59.50 | -54.50 |
| GAUSS-2 | -56.10 | -59.70 | -52.50 |
| ANITSCHKOW | -67.00 | -76.00 | -58.00 |
| GAUSS-4 | -59.50 | -64.79 | -54.21 |
| BATING | -60.70 | -65.80 | -55.60 |
| BERSON | -54.30 | -57.04 | -51.56 |
| EVOPACS | -85.00 | -92.70 | -77.30 |
| DE LA VAL | -40.70 | -45.20 | -36.20 |
| OSLER-1 | -52.70 | -64.20 | -41.20 |
| OSLER-2 | -58.00 | -60.58 | -55.42 |
| BERSON | -62.70 | -65.58 | -59.82 |
| Phase 3 Study of Evolocumab (AMG 145) in Statin-Treated Japanese Patients | -75.90 | -83.54 | -68.26 |
| YUKAWA | -68.60 | -74.48 | -62.72 |
| FOURIER | -59.00 | -60.00 | -58.00 |
| OSLER-1 | -61.00 | -63.00 | -59.00 |
| LAPLACE-2 | -66.00 | -73.00 | -59.00 |

**LDL Mean Difference for Alirocumab**

| **Study** | **LDL Mean Difference** | **Lower 95% CI Limit** | **Upper 95% CI Limit** |
| --- | --- | --- | --- |
| ODYSSEY LONG TERM | -61.90 | -64.30 | -59.50 |
| ODYSSEY DM-DYSLIPIDEMIA | -47.30 | -57.49 | -37.11 |
| ODYSSEY ALTERNATIVE | -30.00 | -36.48 | -23.42 |
| ODYSSEY COMBO II | -29.80 | -34.31 | -25.29 |
| ODYSSEY COMBO I | -45.90 | -52.50 | -39.30 |
| ODYSSEY CHOICE I | -52.70 | -56.42 | -48.98 |
| ODYSSEY EAST | -35.60 | -40.60 | -30.60 |
| ODYSSEY NIPPON | -39.50 | -46.50 | -32.50 |
| ODYSSEY COMBO II | -32.00 | -38.00 | -26.00 |
| Effect of PCSK9 Inhibition by Alirocumab on ... | -72.40 | -78.67 | -66.13 |
| ODYSSEY EAST | -36.90 | -41.80 | -32.00 |
| ODYSSEY OPTIONS I | -44.10 | -52.92 | -35.28 |
| CANTAB | -50.30 | -53.90 | -46.70 |
| ODYSSEY DM-DYSLIPIDEMIA | -37.30 | -43.18 | -31.42 |
| ODYSSEY KT | -63.40 | -71.63 | -55.17 |
| ODYSSEY JAPAN | -34.10 | -38.41 | -29.79 |
| Efficacy and Safety of Alirocumab in Japanese Subjects | -59.60 | -68.03 | -51.17 |
| ODYSSEY OPTIONS II | -50.60 | -58.83 | -42.37 |
| ODYSSEY CHOICE II | -53.50 | -62.71 | -44.29 |
| ODYSSEY MONO | -31.60 | -40.03 | -23.17 |
| ODYSSEY DM-INSULIN | -48.80 | -53.70 | -43.90 |
| ODYSSEY JAPAN | -62.50 | -65.24 | -59.76 |

**LDL Mean Difference for Inclisiran**

| **Study** | **LDL Mean Difference** | **Lower 95% CI Limit** | **Upper 95% CI Limit** |
| --- | --- | --- | --- |
| Orion-10 | -57.00 | -60.15 | -53.85 |
| Orion-11 | -52.70 | -55.75 | -49.65 |
| Orion-1 | -38.40 | -43.60 | -33.20 |
| Orion-9 | -47.90 | -53.39 | -42.41 |

**MACE by Subtype for Evolocumab**

| **Type** | **Author** | **Study** | **Year** | **Event** | **Non-Event** | **Total-Event** | **Event-Placebo** | **Non-Event-Placebo** | **Total-Placebo** | **OddsRatio** | **Lower CI** | **HigherCI** |
| --- | --- | --- | --- | --- | --- | --- | --- | --- | --- | --- | --- | --- |
| Stroke | Sabatine | FOURIER | 2017 | 207 | 13577 | 13784 | 262 | 13518 | 13780 | 0.79 | 0.66 | 0.95 |
| Stroke | Nicholls | GLAGOV | 2016 | 2 | 482 | 484 | 3 | 481 | 484 | 0.67 | 0.11 | 4.00 |
| Stroke | Sabatine | OSLER-1 | 2015 | 3 | 2973 | 2976 | 2 | 1487 | 1489 | 0.75 | 0.13 | 4.49 |
| Stroke | Nissen | GAUSS-3 | 2016 | 0 | 145 | 145 | 0 | 73 | 73 | 0.51 | 0.01 | 25.71 |
| Stroke | Stroes | GAUSS-2 | 2014 | 0 | 102 | 102 | 0 | 205 | 205 | 2.00 | 0.04 | 25.71 |
| Stroke | Robinson | LAPLACE-2 | 2014 | 5 | 1112 | 1117 | 4 | 775 | 779 | 0.87 | 0.23 | 3.25 |
| Myocardial Infarction | Sabatine | FOURIER | 2017 | 468 | 13316 | 13784 | 639 | 13141 | 13780 | 0.72 | 0.64 | 0.82 |
| Myocardial Infarction | Blom | DESCARTES | 2014 | 1 | 598 | 599 | 0 | 302 | 302 | 1.52 | 0.06 | 37.33 |
| Myocardial Infarction | Nissen | GAUSS-3 | 2016 | 1 | 144 | 145 | 1 | 72 | 73 | 0.50 | 0.03 | 8.11 |
| Myocardial Infarction | Nicholls | GLAGOV | 2016 | 10 | 474 | 484 | 14 | 470 | 484 | 0.71 | 0.31 | 1.61 |
| Myocardial Infarction | Sabatine | OSLER-1 | 2015 | 9 | 2967 | 2976 | 5 | 1484 | 1489 | 0.90 | 0.30 | 2.69 |
| Myocardial Infarction | Koren | MENDEL-2 | 2014 | 0 | 306 | 306 | 0 | 154 | 154 | 0.50 | 0.01 | 25.53 |
| Myocardial Infarction | Hirayama | YUKAWA | 2014 | 0 | 105 | 105 | 0 | 102 | 102 | 0.97 | 0.02 | 49.43 |
| Myocardial Infarction | Hirayama | YUKAWA-1 | 2017 | 11 | 135 | 146 | 9 | 64 | 73 | 0.58 | 0.23 | 1.47 |
| Myocardial Infarction | Hirayama | YUKAWA-2 | 2017 | 12 | 212 | 224 | 7 | 106 | 113 | 0.86 | 0.33 | 2.24 |
| Heart Failure | Sabatine | FOURIER | 2017 | 402 | 13382 | 13784 | 408 | 13372 | 13780 | 0.98 | 0.86 | 1.13 |
| Heart Failure | Blom | DESCARTES | 2014 | 1 | 598 | 599 | 0 | 302 | 302 | 1.52 | 0.06 | 37.33 |
| Heart Failure | Sabatine | OSLER-1 | 2015 | 1 | 2975 | 2976 | 1 | 1488 | 1489 | 0.50 | 0.03 | 8.00 |
| Revascularization | Sabatine | FOURIER | 2017 | 759 | 13025 | 13784 | 965 | 12815 | 13780 | 0.77 | 0.7 | 0.85 |
| Revascularization | Nissen | GAUSS-3 | 2016 | 3 | 142 | 145 | 2 | 71 | 73 | 0.75 | 0.12 | 4.59 |
| Revascularization | Stroes | GAUSS-2 | 2014 | 0 | 205 | 205 | 0 | 102 | 102 | 0.50 | 0.01 | 25.23 |
| Revascularization | Nicholls | GLAGOV | 2016 | 50 | 434 | 484 | 66 | 418 | 484 | 0.73 | 0.49 | 1.08 |
| Revascularization | Sabatine | OSLER-1 | 2015 | 15 | 2961 | 2976 | 17 | 1472 | 1489 | 0.44 | 0.22 | 0.88 |
| Revascularization | Sullivan | GAUSS | 2012 | 2 | 60 | 62 | 0 | 32 | 32 | 2.69 | 0.13 | 57.64 |
| Revascularization | Giugliano | LAPLACE-TIMI 57 | 2012 | 2 | 156 | 158 | 1 | 154 | 155 | 1.97 | 0.18 | 22.00 |
| Cardiovascular Mortality | Sabatine | FOURIER | 2017 | 251 | 13533 | 13784 | 240 | 13540 | 13780 | 1.05 | 0.88 | 1.25 |
| Cardiovascular Mortality | Nicholls | GAUSS-3 | 2016 | 3 | 481 | 484 | 4 | 480 | 484 | 0.75 | 0.17 | 3.36 |
| Cardiovascular Mortality | Blom | DESCARTES | 2014 | 2 | 597 | 599 | 0 | 302 | 302 | 2.53 | 0.12 | 52.89 |
| Cardiovascular Mortality | Sullivan | GAUSS | 2012 | 0 | 62 | 62 | 0 | 62 | 62 | 1.00 | 0.02 | 51.19 |
| Cardiovascular Mortality | Stroes | GAUSS-2 | 2014 | 0 | 205 | 205 | 0 | 102 | 102 | 0.50 | 0.01 | 25.32 |
| Cardiovascular Mortality | Nissen | GAUSS-3 | 2016 | 0 | 145 | 145 | 0 | 73 | 73 | 0.51 | 0.01 | 25.71 |
| Cardiovascular Mortality | Robinson | LAPLACE-2 | 2014 | 0 | 1117 | 1117 | 0 | 779 | 779 | 0.70 | 0.01 | 25.71 |
| Cardiovascular Mortality | Giugliano | LAPLACE-TIMI 57 | 2012 | 1 | 473 | 474 | 0 | 155 | 155 | 0.99 | 0.04 | 24.31 |
| Cardiovascular Mortality | Koren | OSLER | 2014 | 1 | 735 | 736 | 0 | 368 | 368 | 1.50 | 0.06 | 36.99 |
| Cardiovascular Mortality | Koren | MENDEL-2 | 2014 | 0 | 308 | 308 | 0 | 308 | 308 | 1.00 | 0.02 | 50.56 |
| Cardiovascular Mortality | Sabatine | OSLER-1 | 2015 | 4 | 2972 | 2976 | 3 | 1486 | 1489 | 0.67 | 0.15 | 2.98 |
| Death (all-cause) | Sabatine | FOURIER | 2017 | 444 | 13340 | 13784 | 426 | 13354 | 13780 | 1.04 | 0.91 | 1.19 |
| Death (all-cause) | Blom | DESCARTES | 2014 | 2 | 597 | 599 | 0 | 302 | 302 | 2.53 | 0.12 | 52.89 |
| Death (all-cause) | Koren | MENDEL-2 | 2014 | 0 | 308 | 308 | 0 | 308 | 308 | 1 | 0.02 | 50.56 |
| Death (all-cause) | Koren | OSLER | 2014 | 1 | 735 | 736 | 2 | 366 | 368 | 0.25 | 0.02 | 2.75 |
| Death (all-cause) | Sabatine | OSLER-1 | 2015 | 4 | 2972 | 2976 | 6 | 1483 | 1489 | 0.33 | 0.09 | 1.18 |

**MACE by Subtype for Alirocumab**

| **Type** | **Author** | **Study** | **Year** | **Event** | **Non-Event** | **Total-Event** | **Event-Placebo** | **Non-Event-Placebo** | **Total-Placebo** | **Odds Ratio** | **LowerCI** | **HigherCI** |
| --- | --- | --- | --- | --- | --- | --- | --- | --- | --- | --- | --- | --- |
| Stroke | Robinson | ODYSSEY LONG TERM | 2015 | 9 | 1541 | 1550 | 2 | 786 | 788 | 2.30 | 0.49 | 10.65 |
| Stroke | Cannon | ODYSSEY COMBO II | 2015 | 1 | 478 | 479 | 1 | 240 | 241 | 0.50 | 0.03 | 8.06 |
| Stroke | Kereiakes | ODYSSEY COMBO I | 2015 | 2 | 205 | 207 | 0 | 107 | 107 | 2.62 | 0.12 | 54.97 |
| Stroke | Han | ODYSSEY EAST | 2020 | 1 | 405 | 406 | 2 | 204 | 206 | 0.25 | 0.02 | 2.79 |
| Stroke | Janik | CANTAB | 2021 | 5 | 1082 | 1087 | 6 | 1078 | 1084 | 0.83 | 0.25 | 2.73 |
| Stroke | Koh | ODYSSEY KT | 2018 | 0 | 97 | 97 | 1 | 101 | 102 | 0.35 | 0.01 | 8.62 |
| Myocardial Infarction | Robinson | ODYSSEY LONG TERM | 2015 | 14 | 1536 | 1550 | 18 | 770 | 788 | 0.39 | 0.19 | 0.79 |
| Myocardial Infarction | Moriarty | ODYSSEY ALTERNATIVE | 2015 | 1 | 125 | 126 | 0 | 124 | 124 | 2.98 | 0.12 | 73.76 |
| Myocardial Infarction | Cannon | ODYSSEY COMBO II | 2015 | 12 | 467 | 479 | 3 | 238 | 241 | 2.04 | 0.57 | 7.29 |
| Myocardial Infarction | Kereiakes | ODYSSEY COMBO I | 2015 | 1 | 206 | 207 | 1 | 106 | 107 | 0.51 | 0.03 | 8.31 |
| Myocardial Infarction | Roth | ODYSSEY CHOICE I | 2016 | 1 | 564 | 565 | 3 | 226 | 229 | 0.13 | 0.01 | 1.29 |
| Myocardial Infarction | Han | ODYSSEY EAST | 2020 | 7 | 399 | 406 | 6 | 200 | 206 | 0.58 | 0.19 | 1.76 |
| Myocardial Infarction | Janik | CANTAB | 2021 | 7 | 1080 | 1087 | 13 | 1071 | 1084 | 0.53 | 0.21 | 1.34 |
| Myocardial Infarction | Koh | ODYSSEY KT | 2018 | 0 | 97 | 97 | 1 | 101 | 102 | 0.35 | 0.01 | 8.62 |
| Myocardial Infarction | Teramoto | ODYSSEY JAPAN | 2016 | 1 | 142 | 143 | 1 | 71 | 72 | 0.50 | 0.03 | 8.11 |
| Heart Failure | Robinson | ODYSSEY LONG TERM | 2015 | 9 | 1541 | 1550 | 3 | 785 | 788 | 1.53 | 0.41 | 5.66 |
| Heart Failure | Cannon | ODYSSEY COMBO II | 2015 | 1 | 478 | 479 | 1 | 240 | 241 | 0.50 | 0.03 | 8.06 |
| Heart Failure | Kereiakes | ODYSSEY COMBO I | 2015 | 0 | 207 | 207 | 1 | 106 | 107 | 0.17 | 0.01 | 4.24 |
| Heart Failure | Han | ODYSSEY EAST | 2020 | 1 | 405 | 406 | 1 | 205 | 206 | 0.51 | 0.03 | 8.13 |
| Heart Failure | Janik | CANTAB | 2021 | 27 | 1060 | 1087 | 21 | 1063 | 1084 | 1.29 | 0.72 | 2.29 |
| Heart Failure | Teramoto | ODYSSEY JAPAN | 2016 | 1 | 142 | 143 | 0 | 72 | 72 | 1.53 | 0.06 | 37.94 |
| Revascularization | Robinson | ODYSSEY LONG TERM | 2015 | 48 | 1502 | 1550 | 24 | 764 | 788 | 1.02 | 0.62 | 1.67 |
| Revascularization | Moriarty | ODYSSEY ALTERNATIVE | 2015 | 3 | 123 | 126 | 1 | 123 | 124 | 3.00 | 0.31 | 29.24 |
| Revascularization | Cannon | ODYSSEY COMBO II | 2015 | 16 | 463 | 479 | 4 | 237 | 241 | 2.05 | 0.68 | 6.19 |
| Revascularization | Kereiakes | ODYSSEY COMBO I | 2015 | 3 | 204 | 207 | 1 | 106 | 107 | 1.56 | 0.16 | 15.17 |
| Revascularization | Han | ODYSSEY EAST | 2020 | 8 | 398 | 406 | 3 | 203 | 206 | 1.36 | 0.36 | 5.18 |
| Revascularization | Koh | ODYSSEY KT | 2018 | 3 | 94 | 97 | 4 | 98 | 102 | 0.78 | 0.17 | 3.59 |
| Revascularization | Teramoto | ODYSSEY JAPAN | 2016 | 2 | 141 | 143 | 1 | 71 | 72 | 1.01 | 0.09 | 11.30 |
| Cardiovascular Mortality | Robinson | ODYSSEY LONG TERM | 2015 | 4 | 1546 | 1550 | 7 | 781 | 788 | 0.29 | 0.08 | 0.99 |
| Cardiovascular Mortality | Cannon | ODYSSEY COMBO II | 2015 | 2 | 477 | 479 | 2 | 239 | 241 | 0.50 | 0.07 | 3.58 |
| Cardiovascular Mortality | Kereiakes | ODYSSEY COMBO I | 2015 | 1 | 206 | 207 | 1 | 106 | 107 | 0.51 | 0.03 | 8.31 |
| Cardiovascular Mortality | Han | ODYSSEY EAST | 2020 | 1 | 405 | 406 | 2 | 204 | 206 | 0.25 | 0.02 | 2.79 |
| Cardiovascular Mortality | Janik | CANTAB | 2021 | 2 | 1085 | 1087 | 5 | 1079 | 1084 | 0.40 | 0.08 | 2.05 |
| Death (all-cause) | Robinson | ODYSSEY LONG TERM | 2015 | 8 | 1542 | 1550 | 10 | 778 | 788 | 0.40 | 0.16 | 1.03 |
| Death (all-cause) | Colhoun | ODYSSEY DM-DYSLIPIDEMIA | 2020 | 1 | 274 | 275 | 0 | 137 | 137 | 1.50 | 0.06 | 37.13 |
| Death (all-cause) | Moriarty | ODYSSEY ALTERNATIVE | 2015 | 0 | 126 | 126 | 0 | 124 | 124 | 0.98 | 0.02 | 49.99 |
| Death (all-cause) | Cannon | ODYSSEY COMBO II | 2015 | 2 | 477 | 479 | 4 | 237 | 241 | 0.25 | 0.05 | 1.37 |
| Death (all-cause) | Kereiakes | ODYSSEY COMBO I | 2015 | 2 | 205 | 207 | 3 | 104 | 107 | 0.34 | 0.06 | 2.06 |
| Death (all-cause) | Roth | ODYSSEY CHOICE I | 2016 | 2 | 571 | 573 | 1 | 228 | 229 | 0.80 | 0.07 | 8.85 |
| Death (all-cause) | Han | ODYSSEY EAST | 2020 | 3 | 403 | 406 | 3 | 203 | 206 | 0.50 | 0.10 | 2.52 |
| Death (all-cause) | Teramoto | ODYSSEY NIPPON | 2019 | 1 | 106 | 107 | 0 | 56 | 56 | 1.59 | 0.06 | 39.71 |
| Death (all-cause) | Bays | ODYSSEY OPTIONS I | 2015 | 0 | 104 | 104 | 2 | 99 | 101 | 0.19 | 0.01 | 4.02 |
| Death (all-cause) | Janik | CANTAB | 2021 | 13 | 1074 | 1087 | 17 | 1067 | 1084 | 0.76 | 0.37 | 1.57 |
| Death (all-cause) | Ray | ODYSSEY DM-DYSLIPIDEMIA | 2018 | 1 | 274 | 275 | 0 | 137 | 137 | 1.50 | 0.06 | 37.13 |
| Death (all-cause) | Koh | ODYSSEY KT | 2018 | 1 | 96 | 97 | 0 | 102 | 102 | 3.19 | 0.13 | 79.17 |
| Death (all-cause) | Teramoto | ODYSSEY JAPAN | 2016 | 0 | 143 | 143 | 0 | 72 | 72 | 0.51 | 0.01 | 25.72 |
| Death (all-cause) | Farnier | ODYSSEY OPTIONS II | 2016 | 0 | 103 | 103 | 1 | 100 | 101 | 0.32 | 0.01 | 8.04 |
| Death (all-cause) | Stroes | ODYSSEY CHOICE II | 2016 | 0 | 173 | 173 | 0 | 58 | 58 | 0.34 | 0.01 | 17.18 |
| Death (all-cause) | Roth | ODYSSEY MONO | 2014 | 0 | 52 | 52 | 0 | 51 | 51 | 0.98 | 0.02 | 50.38 |
| Death (all-cause) | Leiter | ODYSSEY DM-INSULIN | 2017 | 0 | 344 | 344 | 1 | 169 | 170 | 0.16 | 0.01 | 4.05 |
| Death (all-cause) | Ray | ODYSSEY trials (10 - phase 3) | 2016 | 16 | 2302 | 2318 | 13 | 1161 | 1174 | 0.62 | 0.30 | 1.29 |
| Death (all-cause) | Robinson | ODYSSEY trials (4 - phase 3) | 2019 | 14 | 1858 | 1872 | 10 | 941 | 951 | 0.71 | 0.31 | 1.60 |

**MACE by Subtype for Inclisiran**

| **Type** | **Author** | **Study** | **Year** | **Event** | **Non-Event** | **Total-Event** | **Event-Placebo** | **Non-Event-Placebo** | **Total-Placebo** | **Odds Ratio** | **Lower CI** | **Upper CI** |
| --- | --- | --- | --- | --- | --- | --- | --- | --- | --- | --- | --- | --- |
| Stroke | Ray | ORION-10 | 2020 | 11 | 770 | 781 | 7 | 771 | 778 | 1.57 | 0.61 | 4.08 |
| Stroke | Ray | ORION-11 | 2020 | 2 | 809 | 811 | 8 | 796 | 804 | 0.25 | 0.05 | 1.16 |
| Myocardial Infarction | Ray | ORION-10 | 2020 | 20 | 761 | 781 | 18 | 760 | 778 | 1.11 | 0.58 | 2.11 |
| Myocardial Infarction | Ray | ORION-11 | 2020 | 10 | 801 | 811 | 22 | 782 | 804 | 0.44 | 0.21 | 0.94 |
| Cardiovascular Mortality | Ray | ORION-10 | 2020 | 7 | 774 | 781 | 5 | 773 | 778 | 1.40 | 0.44 | 4.42 |
| Cardiovascular Mortality | Ray | ORION-11 | 2020 | 9 | 802 | 811 | 10 | 794 | 804 | 0.89 | 0.36 | 2.20 |
