# Supplementary material for: Proprotein convertase subtilisn/kexin type 9 inhibitors and small interfering RNA therapy for cardiovascular risk reduction: A systematic review and meta-analysis
Source: PLoS One. 2023 Dec 6;18(12):e0295359. doi: 10.1371/journal.pone.0295359 (PMC10699593; doi:10.1371/journal.pone.0295359)
Supplement: S2 File — (DOCX) [file pone.0295359.s003.docx]

S1 Table - Search Strategy

| **Terms** | **Number of results** |
| --- | --- |
| Proprotein convertase subtilisin/kexin type 9 AND *Cholesterol | 3051 |
| Proprotein convertase subtilisin/kexin type 9 AND LDL | 3004 |
| Proprotein convertase subtilisin/kexin type 9 AND Lipid | 3127 |
| Alirocumab AND Cholesterol | 703 |
| Alirocumab AND LDL | 647 |
| Alirocumab AND Lipid | 661 |
| Evolocumab AND Cholesterol | 776 |
| Evolocumab AND LDL | 729 |
| Evolocumab AND Lipid | 732 |
| Inclisiran AND Cholesterol | 200 |
| Inclisiran AND LDL | 187 |
| Inclisiran AND Lipid | 194 |
| PCSK9 inhibitor AND Cholesterol | 2166 |
| PCSK9 inhibitor AND LDL | 2100 |
| PCSK9 inhibitor AND Lipid | 2209 |
| Proprotein convertase subtilisin/kexin type 9 AND Adverse cardiovascular events | 414 |
| Proprotein convertase subtilisin/kexin type 9 AND myocardial infarction | 294 |
| Proprotein convertase subtilisin/kexin type 9 AND stroke | 267 |
| Proprotein convertase subtilisin/kexin type 9 AND mortality | 357 |
| Proprotein convertase subtilisin/kexin type 9 AND cardiovascular mortality | 309 |
| Proprotein convertase subtilisin/kexin type 9 AND heart failure | 84 |
| Proprotein convertase subtilisin/kexin type 9 AND coronary revascularization | 86 |
| Evolocumab AND Adverse cardiovascular events | 206 |
| Evolocumab AND myocardial infarction | 124 |
| Evolocumab AND stroke | 95 |
| Evolocumab AND mortality | 116 |
| Evolocumab AND cardiovascular mortality | 113 |
| Evolocumab AND heart failure | 26 |
| Evolocumab AND coronary revascularization | 50 |
| Alirocumab AND Adverse cardiovascular events | 231 |
| Alirocumab AND myocardial infarction | 89 |
| Alirocumab AND stroke | 87 |
| Alirocumab AND mortality | 102 |
| Alirocumab AND cardiovascular mortality | 97 |
| Alirocumab AND heart failure | 26 |
| Alirocumab AND coronary revascularization | 26 |
| Inclisiran AND Adverse cardiovascular events | 51 |
| Inclisiran AND myocardial infarction | 16 |
| Inclisiran AND stroke | 16 |
| Inclisiran AND mortality | 30 |
| Inclisiran AND cardiovascular mortality | 29 |
| Inclisiran AND heart failure | 11 |
| Inclisiran AND coronary revascularization | 3 |

S2 Table – Demographics for Evolocumab

| N = 22 |  |
| --- | --- |
| Year of publication  2014  2015  2016  2017  2018  2019  2020 | 5 (23.8)  1 (4.8)  1 (4.8)  1 (4.8)  1 (4.6)  9 (42.9)  3 (13.6) |
| Sample size  Mean (SD)  Median (IQR)  Range (Min-Max) | 2012 (5936)  421 (304-968)  (30-27,564) |
| Percent women  Mean (SD)  Median (IQR)  Range (Min-Max) | (n=19)  40.3 (11.9)  41 (30-51)  (18-57) |
| Follow-up period (weeks)  Mean (SD)  Median (IQR)  Range (Min-Max) | 58.7 (87.8)  12 (12-52)  (4-260) |
| Pooled mean difference (weighted)  Mean (95% CI) | -61.1 (-64.8 , -57.4) |

*Categorical data are N(%)

S3 Table – MACE Clinical Trials for Evolocumab

**MACE*: Evolocumab**

| Study or Subgroup | Author | Comparison group exposure | Sample Size | Percent of women (%) | Duration of follow-up | Antibody | | | Comparison group | |
| --- | --- | --- | --- | --- | --- | --- | --- | --- | --- | --- |
|  |  |  |  |  |  | Events | Total | Events | | Total |
| FOURIER | Sabatine et.al. | Placebo | 27564 | 24.6 | 26 months | 1344 | 13784 | 1563 | | 13780 |
| FOURIER-OLE | O’Donoghue et.al. | Placebo to evolocumab | 6635 | 23.3 | 8.4 years | 490 | 3355 | 551 | | 3280 |
| GLAGOV | Nicholls et.al. | Placebo | 968 | 36.6 | 76 weeks | 59 | 484 | 74 | | 484 |
| LAPLACE-TIMI57 | Giugliano et.al. | Placebo with and without ezetimibe | 313 | 51 | 12 weeks | 7 | 158 | 1 | | 155 |
| OSLER-1 | Sabatine et.al. | Standard of care | 4465 | 49 | 48 weeks | 4 | 2976 | 3 | | 1489 |
| OSLER-1 and 2 | Koren et.al. | Standard of care | 1255 | 53 | 5 years | 12 | 1255 | 10 | | 442 |

**Stroke: Evolocumab**

| Study or Subgroup | Author | Comparison group exposure | Sample Size | Percent of women (%) | Duration of follow-up | Antibody | | | Comparison group | |
| --- | --- | --- | --- | --- | --- | --- | --- | --- | --- | --- |
|  |  |  |  |  |  | Events | Total | Events | | Total |
| FOURIER | Sabatine et.al. | Placebo | 27564 | 24.6 | 26 months | 207 | 13784 | 262 | | 13780 |
| FOURIER-OLE | O’Donoghue et.al. | Placebo to evolocumab | 6635 | 23.3 | 8.4 years | 94 | 3355 | 102 | | 3280 |
| GLAGOV | Nicholls et.al. | Placebo | 968 | 36.6 | 76 weeks | 2 | 484 | 3 | | 484 |
| LAPLACE-2 | Robinson et.al. | Placebo and ezetimibe | 1896 |  | 12 weeks | 1 | 1117 | 0 | | 779 |
| OSLER-1 | Sabatine et.al. | Standard of care | 4465 | 49 | 48 weeks | 3 | 2976 | 2 | | 1489 |
| YUKAWA-2 | Kiyosue et.al. | Placebo with or without ezetimibe | 404 | 39.6 | 12 weeks | 0 | 202 | 1 | | 202 |

**Myocardial infarction: Evolocumab**

| Study or Subgroup | Author | Comparison group exposure | Sample Size | Percent of women (%) | Duration of follow-up | Antibody | | Comparison group | |
| --- | --- | --- | --- | --- | --- | --- | --- | --- | --- |
|  |  |  |  |  |  | Events | Total | Events | Total |
| DESCARTES | Blom et.al. | Placebo | 901 | 53 | 52 weeks | 1 | 599 | 0 | 302 |
| FOURIER | Sabatine et.al. | Placebo | 27564 | 24.6 | 26 months | 468 | 13784 | 639 | 13780 |
| FOURIER-OLE | O’Donoghue et.al. | Placebo to evolocumab | 6635 | 23.3 | 8.4 years | 151 | 3355 | 194 | 3280 |
| GAUSS-3 | Nissen et.al. | Ezetimibe + placebo | 218 | 48.6 | 12 weeks | 1 | 145 | 1 | 73 |
| GLAGOV | Nicholls et.al. | Placebo | 968 | 36.6 | 76 weeks | 10 | 484 | 14 | 484 |
| OSLER-1 | Sabatine et.al. | Standard of care | 4465 | 49 | 48 weeks | 9 | 2976 | 5 | 1489 |

**Coronary Revascularization: Evolocumab**

| Study or Subgroup | Author | Comparison group exposure | Sample Size | Percent of women (%) | Duration of follow-up | Antibody | | Comparison group | |
| --- | --- | --- | --- | --- | --- | --- | --- | --- | --- |
|  |  |  |  |  |  | Events | Total | Events | Total |
| FOURIER | Sabatine et.al. | Placebo | 27564 | 24.6 | 26 months | 859 | 13784 | 965 | 13780 |
| FOURIER-OLE | O’Donoghue et.al. | Placebo to evolocumab | 6635 | 23.3 | 8.4 years | 280 | 3355 | 313 | 3280 |
| GAUSS | Sullivan et.al. | Placebo + ezetimibe | 160 | 64 |  | 2 | 62 | 0 | 32 |
| GAUSS-3 | Nissen et.al. | Ezetimibe + placebo | 218 | 48.6 | 12 weeks | 3 | 145 | 2 | 73 |
| GLAGOV | Nicholls et.al. | Placebo | 968 | 36.6 | 76 weeks | 50 | 484 | 66 | 484 |
| LAPLACE-TIMI57 | Giugliano et.al. | Placebo with and without ezetimibe | 313 | 51 | 12 weeks | 2 | 158 | 1 | 155 |
| OSLER-1 | Sabatine et.al. | Standard of care | 4465 | 49 | 48 weeks | 15 | 2976 | 17 | 1489 |
| YUKAWA-1 | Hirayama et.al. | Placebo with or without ezetimibe | 207 | 37.1 | 12 weeks | 0 | 105 | 2 | 102 |

**Acute Heart Failure: Evolocumab**

| Study or Subgroup | Author | Comparison group exposure | Sample Size | Percent of women (%) | Duration of follow-up | Antibody | | Comparison group | | |
| --- | --- | --- | --- | --- | --- | --- | --- | --- | --- | --- |
|  |  |  |  |  |  | Events | Total | Events | Total |  |
| DESCARTES | Blom et.al. | Placebo | 901 | 53 | 52 weeks | 1 | 599 | 0 | 302 |  |
| FOURIER | Sabatine et.al. | Placebo | 27564 | 24.6 | 26 months | 402 | 13784 | 408 | 13780 |  |
| FOURIER-OLE | O’Donoghue et.al. | Placebo to evolocumab | 6635 | 23.3 | 8.4 years | 151 | 3355 | 194 | 3280 |  |
| OSLER-1 | Sabatine et.al. | Standard of care | 4465 | 49 | 48 weeks | 1 | 2976 | 1 | 1489 |  |

**Cardiovascular Mortality: Evolocumab**

| Study or Subgroup | Author | Comparison group exposure | Sample Size | Percent of women (%) | Duration of follow-up | Antibody | | Comparison group | |
| --- | --- | --- | --- | --- | --- | --- | --- | --- | --- |
|  |  |  |  |  |  | Events | Total | Events | Total |
| DESCARTES | Blom et.al. | Placebo | 901 | 53 | 52 weeks | 2 | 599 | 0 | 302 |
| FOURIER | Sabatine et.al. | Placebo | 27564 | 24.6 | 26 months | 251 | 13784 | 240 | 13780 |
| FOURIER-OLE | O’Donoghue et.al. | Placebo to evolocumab | 6635 | 23.3 | 8.4 years | 107 | 3355 | 138 | 3280 |
| GLAGOV | Nicholls et.al. | Placebo | 968 | 36.6 | 76 weeks | 3 | 484 | 4 | 484 |
| LAPLACE-2 | Robinson et.al. | Placebo and ezetimibe | 1896 |  | 12 weeks | 0 | 1117 | 1 | 779 |
| LAPLACE-TIMI57 | Giugliano et.al. | Placebo with and without ezetimibe | 313 | 51 | 12 weeks | 1 | 158 | 0 | 155 |
| OSLER-1 | Sabatine et.al. | Standard of care | 4465 | 49 | 48 weeks | 4 | 2976 | 3 | 1489 |

**All-cause Mortality: Evolocumab**

| Study or Subgroup | Author | Sample Size | Percent of women (%) | Duration of follow-up | Antibody | | Comparison group | |
| --- | --- | --- | --- | --- | --- | --- | --- | --- |
|  |  |  |  |  | Events | Total | Events | Total |
| DESCARTES | Blom et.al. | 901 | 53 | 52 weeks | 2 | 599 | 0 | 302 |
| FOURIER | Sabatine et.al. | 27564 | 24.6 | 26 months | 444 | 13784 | 426 | 13780 |
| FOURIER-OLE | O’Donoghue et.al. | 6635 | 23.3 | 8.4 years | 338 | 3355 | 334 | 3280 |
| GLAGOV | Nicholls et.al. | 968 | 36.6 | 76 weeks | 3 | 484 | 4 | 484 |
| LAPLACE-2 | Robinson et.al. | 1896 |  | 12 weeks | 0 | 1117 | 1 | 779 |
| LAPLACE-TIMI57 | Giugliano et.al. | 313 | 51 | 12 weeks | 1 | 158 | 0 | 155 |
| OSLER-1 | Sabatine et.al. | 4465 | 49 | 48 weeks | 4 | 2976 | 6 | 1489 |

* MACE: major adverse cardiac events (composite of myocardial infarction, cardiovascular death, stroke, coronary revascularization, unstable angina)

CV: cardiovascular

UA: unstable angina

HLD: hyperlipidemia

LDL-C: low density lipoprotein cholesterol

S4 Table – MACE Clinical Trials for Alirocumab

**Stroke: Alirocumab**

| Study or Subgroup | Author | Comparison group exposure | Sample Size | Percent of women (%) | Duration of follow-up | Antibody | | Comparison group | |
| --- | --- | --- | --- | --- | --- | --- | --- | --- | --- |
|  |  |  |  |  |  | Events | Total | Events | Total |
| ODESSY OUTCOMES | Schwartz et.al. | Placebo | 18924 | 25 | 26 months | 111 | 9462 | 152 | 9310 |
| ODESSY LONG TERM | Robinson et.al. | Placebo | 2341 | 38.8 | 24 weeks | 9 | 1550 | 2 | 788 |
| ODESSY COMBO II | Cannon et.al. | Ezetimibe | 720 | 26.4 | 24 weeks | 1 | 479 | 1 | 241 |
| ODESSY COMBO I | Kereiakes et.al. | Placebo | 316 | 34.2 | 52 weeks | 2 | 207 | 0 | 107 |
| ODESSY EAST | Han et.al. | Ezetimibe | 615 | 25 | 24 weeks | 1 | 406 | 2 | 204 |
| CANTAB | Janik et.al. | Placebo | 2176 | 41.8 | 96 weeks | 5 | 1087 | 6 | 1084 |
| ODYSSEY KT | Koh et.al. | Placebo | 199 | 17.6 | 24 weeks | 0 | 97 | 1 | 102 |

**Myocardial Infarction: Alirocumab**

| Study or Subgroup | Author | Comparison group exposure | Sample Size | Percent of women (%) | Duration of follow-up | Antibody | | Comparison group | |
| --- | --- | --- | --- | --- | --- | --- | --- | --- | --- |
|  |  |  |  |  |  | Events | Total | Events | Total |
| ODESSY OUTCOMES | Schwartz et.al. | Placebo | 18924 | 25 | 26 months | 626 | 9462 | 722 | 9310 |
| ODESSY LONG TERM | Robinson et.al. | Placebo | 2341 | 38.8 | 24 weeks | 14 | 1550 | 18 | 788 |
| ODDESY ALTERNATIVE | Moriarty et.al. | Ezetimibe or atorvastatin monotherapy | 314 | 45.4 | 24 weeks | 1 | 126 | 0 | 124 |
| ODESSY COMBO II | Cannon et.al. | Ezetimibe | 720 | 26.4 | 24 weeks | 12 | 479 | 3 | 241 |
| ODESSY COMBO I | Kereiakes et.al. | Placebo | 316 | 34.2 | 52 weeks | 1 | 207 | 1 | 107 |
| ODESSY EAST | Han et.al. | Ezetimibe | 615 | 25 | 24 weeks | 7 | 406 | 6 | 204 |
| CANTAB | Janik et.al. | Placebo | 2176 | 41.8 | 96 weeks | 7 | 1087 | 13 | 1084 |
| ODYSSEY KT | Koh et.al. | Placebo | 199 | 17.6 | 24 weeks | 0 | 97 | 1 | 102 |
| ODYSSEY JAPAN | Teramoto et.al. |  | 216 | 39.4 | 52 weeks | 1 | 143 | 1 | 72 |

**Heart Failure: Alirocumab**

| Study or Subgroup | Author | Comparison group exposure | Sample Size | Percent of women (%) | Duration of follow-up | Antibody | | Comparison group | |
| --- | --- | --- | --- | --- | --- | --- | --- | --- | --- |
|  |  |  |  |  |  | Events | Total | Events | Total |
| ODESSY OUTCOMES | Schwartz et.al. | Placebo | 18924 | 25 | 26 months | 176 | 9462 | 179 | 9310 |
| ODESSY LONG TERM | Robinson et.al. | Placebo | 2341 | 38.8 | 24 weeks | 9 | 1550 | 3 | 788 |
| ODESSY COMBO II | Cannon et.al. | Ezetimibe | 720 | 26.4 | 24 weeks | 1 | 479 | 1 | 241 |
| ODESSY COMBO I | Kereiakes et.al. | Placebo | 316 | 34.2 | 52 weeks | 0 | 207 | 1 | 107 |
| ODESSY EAST | Han et.al. | Ezetimibe | 615 | 25 | 24 weeks | 1 | 406 | 1 | 204 |
| CANTAB | Janik et.al. | Placebo | 2176 | 41.8 | 96 weeks | 27 | 1087 | 21 | 1084 |
| ODYSSEY KT | Koh et.al. | Placebo | 199 | 17.6 | 24 weeks | 0 | 97 | 1 | 102 |
| ODYSSEY JAPAN | Teramoto et.al. |  | 216 | 39.4 | 52 weeks | 1 | 143 | 0 | 72 |

**Coronary Revascularization: Alirocumab**

| Study or Subgroup | Author | Comparison group exposure | Sample Size | Percent of women (%) | Duration of follow-up | Antibody | | Comparison group | |
| --- | --- | --- | --- | --- | --- | --- | --- | --- | --- |
|  |  |  |  |  |  | Events | Total | Events | Total |
| ODESSY OUTCOMES | Schwartz et.al. | Placebo | 18924 | 25 | 26 months | 731 | 9462 | 828 | 9310 |
| ODESSY LONG TERM | Robinson et.al. | Placebo | 2341 | 38.8 | 24 weeks | 48 | 1550 | 24 | 788 |
| ODESSY COMBO II | Cannon et.al. | Ezetimibe | 720 | 26.4 | 24 weeks | 16 | 479 | 4 | 241 |
| ODESSY COMBO II | El Shahawyet.al. | Ezetimibe | 720 | 26.4 | 2 years | 21 | 479 | 7 | 241 |
| ODESSY COMBO I | Kereiakes et.al. | Placebo | 316 | 34.2 | 52 weeks | 3 | 207 | 1 | 107 |
| ODESSY EAST | Han et.al. | Ezetimibe | 615 | 25 | 24 weeks | 8 | 406 | 3 | 204 |
| ODYSSEY KT | Koh et.al. | Placebo | 199 | 17.6 | 24 weeks | 3 | 97 | 4 | 102 |
| ODYSSEY JAPAN | Teramoto et.al. |  | 216 | 39.4 | 52 weeks | 2 | 143 | 1 | 72 |

**Cardiovascular Mortality: Alirocumab**

| Study or Subgroup | Author | Comparison group exposure | Sample Size | Percent of women (%) | Duration of follow-up | Antibody | | Comparison group | |
| --- | --- | --- | --- | --- | --- | --- | --- | --- | --- |
|  |  |  |  |  |  | Events | Total | Events | Total |
| ODESSY OUTCOMES | Schwartz et.al. | Placebo | 18924 | 25 | 26 months | 240 | 9462 | 271 | 9310 |
| ODESSY LONG TERM | Robinson et.al. | Placebo | 2341 | 38.8 | 24 weeks | 4 | 1550 | 7 | 788 |
| ODESSY COMBO II | Cannon et.al. | Ezetimibe | 720 | 26.4 | 24 weeks | 2 | 479 | 2 | 241 |
| ODESSY COMBO II | El Shahawyet.al. | Ezetimibe | 720 | 26.4 | 2 years | 4 | 479 | 2 | 241 |
| ODESSY COMBO I | Kereiakes et.al. | Placebo | 316 | 34.2 | 52 weeks | 1 | 207 | 1 | 107 |
| ODESSY EAST | Han et.al. | Ezetimibe | 615 | 25 | 24 weeks | 1 | 406 | 2 | 204 |
| CANTAB | Janik et.al. | Placebo | 2176 | 41.8 | 96 weeks | 2 | 1087 | 5 | 1084 |
| ODYSSEY KT | Koh et.al. | Placebo | 199 | 17.6 | 24 weeks | 3 | 97 | 4 | 102 |
| ODYSSEY JAPAN | Teramoto et.al. |  | 216 | 39.4 | 52 weeks | 2 | 143 | 1 | 72 |

**All-cause mortality: Alirocumab**

| Study or Subgroup | Author | Comparison group exposure | Sample Size | Percent of women (%) | Duration of follow-up | Antibody | | Comparison group | |
| --- | --- | --- | --- | --- | --- | --- | --- | --- | --- |
|  |  |  |  |  |  | Events | Total | Events | Total |
| ODESSY OUTCOMES | Schwartz et.al. | Placebo | 18924 | 25 | 26 months | 334 | 9462 | 392 | 9310 |
| ODESSY LONG TERM | Robinson et.al. | Placebo | 2341 | 38.8 | 24 weeks | 8 | 1550 | 10 | 788 |
| ODESSY COMBO II | Cannon et.al. | Ezetimibe | 720 | 26.4 | 24 weeks | 2 | 479 | 4 | 241 |
| ODESSY COMBO II | El Shahawyet.al. | Ezetimibe | 720 | 26.4 | 2 years | 6 | 479 | 6 | 241 |
| ODESSY COMBO I | Kereiakes et.al. | Placebo | 316 | 34.2 | 52 weeks | 2 | 207 | 3 | 107 |
| ODESSY CHOICE I | Roth et.al. |  | 803 | 42.4 | 48 weeks | 2 | 573 | 1 | 229 |
| ODESSY EAST | Han et.al. | Ezetimibe | 615 | 25 | 24 weeks | 3 | 406 | 3 | 204 |
| ODYSSEY DM- DYSLIPIDEMIA | Colhoun et.al. | Standard of care | 413 | 50 | 24 weeks | 1 | 275 | 0 | 137 |
| ODYSSEY DM- DYSLIPIDEMIA | Ray et.al. | Standard of care | 413 | 47.7 | 24 weeks | 1 | 275 | 0 | 137 |
| ODYSSEY DM – INSULIN | Leiter et.al. | Placebo | 517 | 45 | 24 weeks | 0 | 334 | 1 | 170 |
| ODYSSEY NIPPON | Teramoto et.al. | Placebo | 163 | 36.8 | 12 weeks | 1 | 107 | 0 | 56 |
| ODYSSEY OPTIONS I | Bays et.al. | Atorvastatin or rosuvastatin | 355 | 35 | 24 weeks | 0 | 104 | 2 | 101 |
| ODYSSEY Trials (Phase 3) | Ray et.al. | Ezetimibe, placebo | 4983 | 38 | Variable | 16 | 2318 | 13 | 1161 |
| ODYSSEY Trials (Phase 4) | Robinson et.al. | Various doses of arilocumab | 3650 | 38 | Variable | 14 | 1872 | 10 | 941 |
| CANTAB | Janik et.al. | Placebo | 2176 | 41.8 | 96 weeks | 13 | 1087 | 17 | 1084 |
| ODYSSEY KT | Koh et.al. | Placebo | 199 | 17.6 | 24 weeks | 1 | 97 | 0 | 102 |

S5 Table – MACE Clinical Trials for Inclisiran

**MACE: Inclisiran**

| Study or Subgroup | Author | Comparison group exposure | Sample Size | Percent of women (%) | Duration of follow-up | Antibody | | Comparison group | |
| --- | --- | --- | --- | --- | --- | --- | --- | --- | --- |
|  |  |  |  |  |  | Events | Total | Events | Total |
| ORION-1 | Kausik et.al. | Placebo | 483 | 33.1 | 180 days | 2 | 370 | 0 | 113 |
| ORION-9 | Raal et.al. | Placebo | 482 | 53 | 8.4 years | 1 | 241 | 0 | 240 |
| ORION-10 | Ray et.al. | Placebo | 1559 | 28 | 540 days | 38 | 78*1* | 30 | 778 |
| ORION-11 | Ray et.al. | Placebo | 1615 | 28 | 450 days | 21 | 811 | 40 | 764 |

S6 Table – Meta-regression adjusting for age and percent women

PCSK9i meta-regression results for LDL mean difference

Table 1. Alirocumab

| Covariate | β coefficient (95% CI) | *p*-value |
| --- | --- | --- |
| Mean Age | 1.96 (-0.89 , 4.82) | 0.18 |
| Percent women | -0.35 (-0.89 , 0.20) | 0.22 |

Residual heterogeneity:

t^2^ = 134.1

I^2^ = 95.48%

H^2^ = 23.49

Table 2. Evolocumab

| Covariate | β coefficient (95% CI) | *p*-value |
| --- | --- | --- |
| Mean Age | -0.62 (-2.07 , 0.82) | 0.39 |
| Percent women | -0.33 (-0.67 , 0.01) | 0.059 |

Residual heterogeneity:

t^2^ = 71.96

I^2^ = 96.91%

H^2^ = 32.4

Table 3. Inclisiran

| Covariate | β coefficient (95% CI) | *p*-value |
| --- | --- | --- |
| Mean Age | 0.27 (-13.57 , 14.11) | 0.97 |
| Percent women | 0.32 (-5.31 , 5.94) | 0.91 |

Residual heterogeneity:

t^2^ = 169.2

I^2^ = 96.76

H^2^ = 30.84

S1 Figure – Funnel plot of LDL mean difference for Alirocumab and Evolocumab trials

*Egger’s test *p*-value = 0.71

S2 Figure - Funnel plot of LDL mean difference for Inclisiran trials

*Egger’s test *p*-value = 0.046

S3 Figure – Funnel plot of MACE for Alirocumab and Evolocumab trials

*Egger’s test *p*-value = 0.09

S4 Figure - Funnel plot of MACE for Inclisiran trials

**MACE Endpoint definitions across studies**

- **Cardiovascular death** includes death resulting from an acute myocardial infarction (MI), sudden cardiac death, death due to heart failure (HF), death due to stroke, death due to cardiovascular (CV) procedures, death due to CV hemorrhage, and death due to other CV causes.
- **Myocardial Infarction (MR)** requires the combination of:
  - Evidence of myocardial necrosis (either changes in cardiac biomarkers or post-mortem pathological findings); and
  - Supporting information derived from the clinical presentation, electrocardiographic changes, or the results of myocardial or coronary artery imaging
    - Biomarker Elevations – troponins are preferred. CK-MB should be used if troponins are not available, and total CK may be used in the absence of CK-MB and troponin.
    - ECG
      - New ST elevation at the J point in two contiguous leads with the cutpoints: ≥ 0.1 mV in all leads other than leads V2-V3 where the following cut-points apply: ≥ 0.2 mV in men ≥ 40 years (≥ 0.25 mV in men < 40 years) or ≥ 0.15 mV in women.
      - New horizontal or down-sloping ST depression ≥ 0.05 mV in two contiguous leads and/or new T inversion ≥ 0.1 mV in two contiguous leads with prominent R wave or R/S ratio > 1.
      - Q-Wave
        - Any Q-wave in leads V2-V3 ≥ 0.02 seconds or QS complex in leads V2 and V3 o Q-wave ≥ 0.03 seconds and ≥ 0.1 mV deep or QS complex in leads I, II, aVL, aVF, or V4-V6 in any two leads of a contiguous lead grouping (I, aVL; V1-V6; II, III, and aVF)
    - STEMI vs NSTEMI
- **Coronary Revascularization**
  - **Percutaneous Coronary Intervention (PCI):** Placement of an angioplasty guide wire, balloon, or other device (e.g., stent, atherectomy catheter, brachytherapy delivery device, or thrombectomy catheter) into a native coronary artery or coronary artery bypass graft for the purpose of mechanical coronary revascularization. In the assessment of the severity of coronary lesions with the use of intravascular ultrasound, CFR, or FFR, insertion of a guide wire will NOT be considered PCI.
    - **Elective:** The procedure can be performed on an outpatient basis or during a subsequent hospitalization without significant risk of myocardial infarction (MI) or death. For stable in-patients, the procedure is being performed during this hospitalization for convenience and ease of scheduling and **NOT** because the patient's clinical situation demands the procedure prior to discharge.
    - **Urgent:** The procedure should be performed on an inpatient basis and prior to discharge because of significant concerns that there is risk of myocardial ischemia, MI, and/or death. Patients who are outpatients or in the emergency department at the time that the cardiac catheterization is requested would warrant hospital admission based on their clinical presentation.
    - **Emergency:** The procedure should be performed as soon as possible because of substantial concerns that ongoing myocardial ischemia and/or MI could lead to death. "As soon as possible" refers to a patient who is of sufficient acuity that one would cancel a scheduled case to perform this procedure immediately in the next available room during business hours, or one would activate the on-call team were this to occur during off-hours.
    - **Salvage:** The procedure is a last resort. The patient is in cardiogenic shock when the PCI begins (i.e., the time at which the first guide wire or intracoronary device is introduced into a coronary artery or bypass graft for the purpose of mechanical revascularization) **OR** within the last ten minutes prior to the start of the case or during the diagnostic portion of the case, the patient has also received chest compressions or has been on unanticipated circulatory support (e.g., intra-aortic balloon pump, extracorporeal mechanical oxygenation, or cardiopulmonary support).
- **Heart Failure**
  - A Heart Failure Event includes hospitalization for heart failure and may include urgent outpatient visits. HF hospitalizations should remain delineated from urgent visits. If urgent visits are included in the HF event endpoint, the number of urgent visits needs to be explicitly presented separately from the hospitalizations.
  - A Heart Failure Hospitalization is defined as an event that meets ALL of the following criteria:
    - The patient is admitted to the hospital with a primary diagnosis of HF
    - The patient’s length-of-stay in hospital extends for at least 24 hours
    - The patient exhibits documented new or worsening symptoms due to HF on presentation, including at least ONE of the following: a) Dyspnea (dyspnea with exertion, dyspnea at rest, orthopnea, paroxysmal nocturnal dyspnea) b) Decreased exercise tolerance c) Fatigue d) Other symptoms of worsened end-organ perfusion or volume overload
    - The patient has objective evidence of new or worsening HF, consisting of at least TWO physical examination findings a) OR one physical examination finding and at least ONE laboratory criterion
    - The patient receives initiation or intensification of treatment specifically for HF, including at least ONE of the following: a) Augmentation in oral diuretic therapy b) Intravenous diuretic, inotrope, or vasodilator therapy c) Mechanical or surgical intervention, including 1) Mechanical circulatory support (e.g., intra-aortic balloon pump, ventricular assist device) 2) Mechanical fluid removal (e.g., ultrafiltration, hemofiltration, dialysis)
- **Cerebrovascular Events**
  - The distinction between a Transient Ischemic Attack and an Ischemic Stroke is the presence of infarction. Persistence of symptoms is an acceptable indicator of acute infarction.
  - **Transient ischemic attack (TIA)** is defined as a transient episode of focal neurological dysfunction caused by brain, spinal cord, or retinal ischemia, without acute infarction.
  - **Stroke** is defined as an acute episode of focal or global neurological dysfunction caused by brain, spinal cord, or retinal vascular injury as a result of hemorrhage or infarction.
    - Classification:
    - 1. Ischemic Stroke
      - Ischemic stroke is defined as an acute episode of focal cerebral, spinal, or retinal dysfunction caused by infarction of central nervous system tissue.
      - Hemorrhage may be a consequence of ischemic stroke. In this situation, the stroke is an ischemic stroke with hemorrhagic transformation and not a hemorrhagic stroke.
    - 2. Hemorrhagic Stroke
      - Hemorrhagic stroke is defined as an acute episode of focal or global cerebral or spinal dysfunction caused by intraparenchymal, intraventricular, or subarachnoid hemorrhage
    - 3. Undetermined Stroke
      - Undetermined stroke is defined as an acute episode of focal or global neurological dysfunction caused by presumed brain, spinal cord, or retinal vascular injury as a result of hemorrhage or infarction but with insufficient information to allow categorization as 1 or 2.
    - Disability should be measured by a reliable and valid scale in all cases, typically at each visit and 90 days after the event.
  - **Stroke Disability** should be measured by a reliable and valid scale in all cases, typically at each visit and 90 days after the event.
